# Nitric oxide activatable photosensitizer accompanying extremely elevated two-photon absorption for efficient fluorescence imaging and photodynamic therapy†
†Electronic supplementary information (ESI) available: Experimental details of synthesis, characterization and supplementary figures. See DOI: 10.1039/c7sc04044j


**DOI:** 10.1039/c7sc04044j

**Published:** 2017-11-27

**Authors:** Wenbo Hu, Meng Xie, Hui Zhao, Yufu Tang, Song Yao, Tingchao He, Chuanxiang Ye, Qi Wang, Xiaomei Lu, Wei Huang, Quli Fan

**Affiliations:** a Key Laboratory for Organic Electronics and Information Displays , Institute of Advanced Materials (IAM) , Jiangsu National Synergetic Innovation Center for Advanced Materials (SICAM) , Nanjing University of Posts & Telecommunications , Nanjing 210023 , China . Email: iamqlfan@njupt.edu.cn; b Key Laboratory of Flexible Electronics (KLOFE) , Institute of Advanced Materials (IAM) , Jiangsu National Synergetic Innovation Center for Advanced Materials (SICAM) , Nanjing Tech University (NanjingTech) , Nanjing 211816 , China . Email: wei-huang@njtech.edu.cn; c Key Laboratory of Optoelectronic Devices and Systems of Ministry of Education and Guangdong Province , College of Physics Science & Technology , Shenzhen University , Shenzhen 518060 , China; d Shaanxi Institute of Flexible Electronics (SIFE) , Northwestern Polytechnical University (NPU) , Xi’an 710072 , China

## Abstract

Elevated nitric oxide (NO) levels perform an important pathological role in various inflammatory diseases. Developing NO-activatable theranostic materials with a two-photon excitation (TPE) feature is highly promising for precision imaging and therapy, but constructing such materials is still a tremendous challenge. Here, we present the first example of a NO-activatable fluorescent photosensitizer (**DBB-NO**) accompanying extremely NO-elevated two-photon absorption (TPA) for efficient fluorescence imaging and photodynamic therapy (PDT). Upon responding to NO, **DBB-NO** shows not only a remarkably enhanced fluorescence quantum yield (*Φ*_F_, 0.17% *vs.* 9.3%) and singlet oxygen quantum yield (*Φ*_Δ_, 1.2% *vs.* 82%) but also an extremely elevated TPA cross-section (*δ*, 270 *vs.* 2800 GM). Simultaneous enhancement of *Φ*_Δ_, *Φ*_F_ and *δ* allows unprecedented two-photon fluorescence brightness (*δ* × *Φ*_F_ = 260.4 GM) and two-photon PDT (TP-PDT) efficiency (*δ* × *Φ*_Δ_ = 2296 GM) which precedes the value for a commercial two-photon photosensitizer by two orders of magnitude. With these merits, the proof-of-concept applications of NO-activatable two-photon fluorescence imaging and TP-PDT in activated macrophages (in which NO is overproduced) were readily realized. This work may open up many opportunities for constructing two-photon theranostic materials with other pathological condition-activatable features for precise theranostics.

## Abstract 2

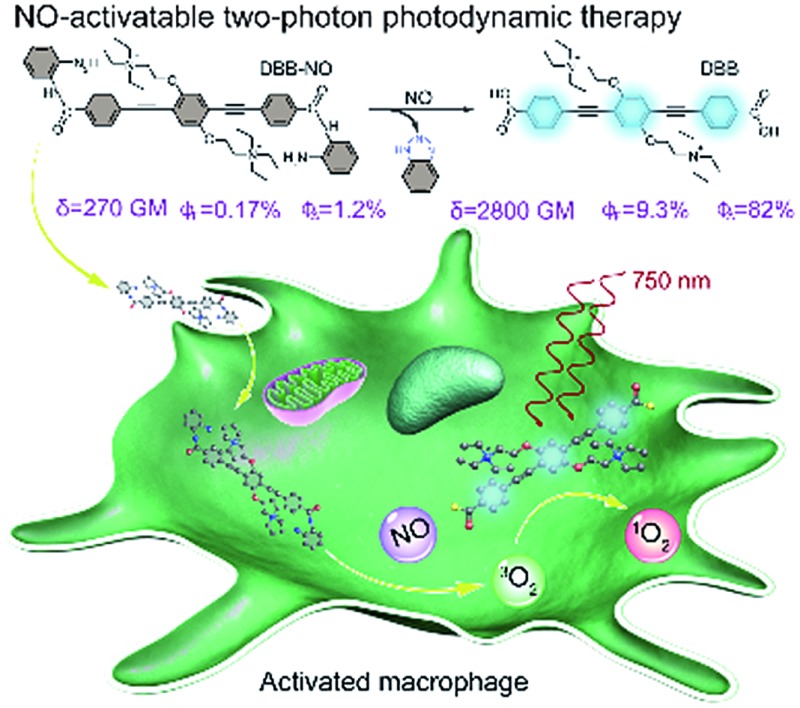
A nitric oxide (NO) activatable photosensitizer was constructed for efficient fluorescence imaging and photodynamic therapy.

## Introduction

Nitric oxide (NO) is an endogenous gaseous cellular messenger molecule which plays a pivotal role in the cardiovascular, nervous, and immune systems for various physiological functions.^1^ However, an elevated NO level will induce pathological conditions including glaucoma, age-related macular degeneration, and autoimmune and inflammatory diseases.^2^ This feature makes NO a promising target to develop NO-activatable theranostic materials not only for deep insights into the pathological activities of NO, but also for the innovative therapeutic approach to precisely treat these diseases.^3^ Despite considerable efforts centred on the design of a NO-activatable fluorescent probe to monitor the NO concentration produced either *in vitro* or *in vivo*,^4^ NO-activatable therapeutic materials have heretofore never been reported.

Photodynamic therapy (PDT), whereby a cytotoxic singlet oxygen (^1^O_2_) is generated by a photosensitizer (PS), is a clinically approved yet non-invasive therapeutic approach.^5^ A particular interest of novel PDT is to develop a fluorescent PS (FPS) as a theranostic material for simultaneous fluorescence imaging and PDT,^6^ which intrinsically circumvents the complicated fabrication procedure of the traditional theranostic materials. Recently, FPSs that can only be activated in pathological conditions have become very appealing for precise imaging and selective killing of target cells.^7^ The design of the activatable FPS generally requires the conjugation of a stimulus-responsive quencher with the FPS to switch off its fluorescence and PDT ability *via* intramolecular photoinduced electron transfer (PeT).^7^ To ensure the PeT process, the energy level alignment between the quencher and the FPS is of particular importance. However, the energy level of an available NO-responsive moiety, such as typical *o*-phenylenediamine (OPD), can align only to those FPSs with ultraviolet (UV) or visible light absorption,^8^ which is apparently unfavorable for theranostics in deep tissues. One attractive approach to address this problem is to make use of a two-photon absorbing FPS (TP-FPS),^9^ which can not only ensure the required energy level alignment but also permit a better penetration depth and spatial selectivity that is unattainable with traditional one-photon excitation.^10^ Although conceptually impressively, the ultralow two-photon absorption cross-section (*δ*) of the available TP-FPS with values of 1–100 GM (GM: Goeppert–Mayer units) makes two-photon excited PDT (TP-PDT) in practical applications more challenging.^11^

**Scheme 1:**
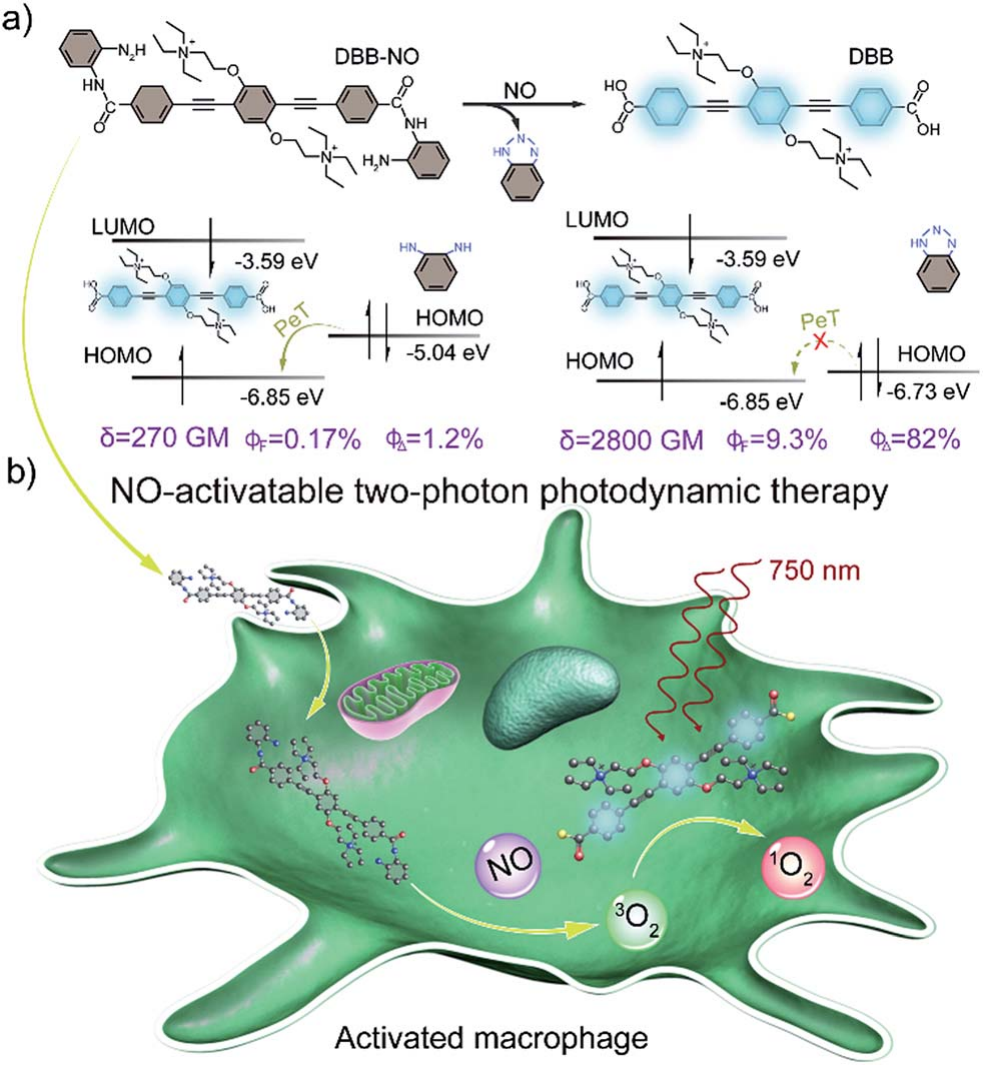
(a) Design principle and molecular structure of the NO-activatable TP-FPS (**DBB-NO**). LUMO, lowest unoccupied molecular orbital; HOMO, highest occupied molecular orbital; PeT, photoinduced electron transfer; *δ*, TPA cross-section; *Φ*_F_, absolute fluorescence quantum yield; *Φ*_Δ_, singlet oxygen quantum yield. (b) Schematic illustration of the NO-activatable TP-FPS for TP-imaging and TP-PDT in an activated macrophage (in which NO is upregulated).

Herein, we present a rational design to construct a NO-activatable TP-FPS with extremely NO-elevated *δ* to realize efficient TP-imaging and TP-PDT (Scheme 1). We coupled a zwitterionic bis(phenylethynyl)benzene derivative (DBB) with OPD through a typical amidation reaction to form the target TP-FPS (**DBB-NO**). Such a molecule design depends on the following considerations. First, the bis(phenylethynyl)benzene-based conjugated backbone, owing to its excellent absorption in the UV region,^12^ ensures a desirable energy level alignment with OPD for efficient PeT to maintain quenched fluorescence and PDT. Second, our previous work demonstrates that bis(phenylethynyl)benzene with zwitterionic groups exhibits an ultrahigh *δ* and ^1^O_2_ quantum yield (*Φ*_Δ_).^13^ Therefore, we envision that the release of zwitterionic DBB after the cleavage of OPD from **DBB-NO** by NO can simultaneously switch on the *δ* and *Φ*_Δ_, both of which are beneficial for realizing efficient TP-PDT. Third, coupling an amino group of OPD with the carboxyl group within DBB can improve the selectivity of **DBB-NO** to NO, effectively avoiding the “false positive” fluorescence signals or unexpected phototoxicity toward healthy cells. Finally, **DBB-NO** shows an extremely elevated *δ* (270 *vs.* 2800 GM) and *Φ*_Δ_ (2.2% *vs.* 82%) as well as a slightly enhanced fluorescence quantum yield (*Φ*_F_, 0.17% *vs.* 9.3%) upon responding to NO, thus enabling the first example of the proof-of-concept applications of NO-activatable TP-imaging and TP-PDT in activated macrophages (in which NO is overproduced) and even in a more complex inflamed mouse model.

## Results and discussion

### Design and characterizations

The synthetic route to the NO-activatable TP-FPS of **DBB-NO** was presented in Scheme S1.† Built on the abovementioned considerations, we first synthesized the zwitterionic **DBB** structure with an end-capped carboxyl group (Scheme 1) as a potential TP-FPS. Then to avoid the unwanted response of the OPD-based probe toward other competitive species including ascorbic acid (AA), dehydroascorbic acid (DHA) and methylglyoxal (MGO) that have been observed in other reports,4a,^14^ the carboxyl group of **DBB** was directly coupled with an amino group of OPD through a typical amidation reaction. This is because OPD *via* this kind of connection will lose reaction ability with other competitive species.^15^ The detailed synthetic procedures, ^1^H NMR, ^13^C NMR, and high-resolution mass spectrum (HRMS) are given in ESI (Scheme S1 and Fig. S1–S9†).

### Spectral response of **DBB-NO** to NO

**Fig. 1:**
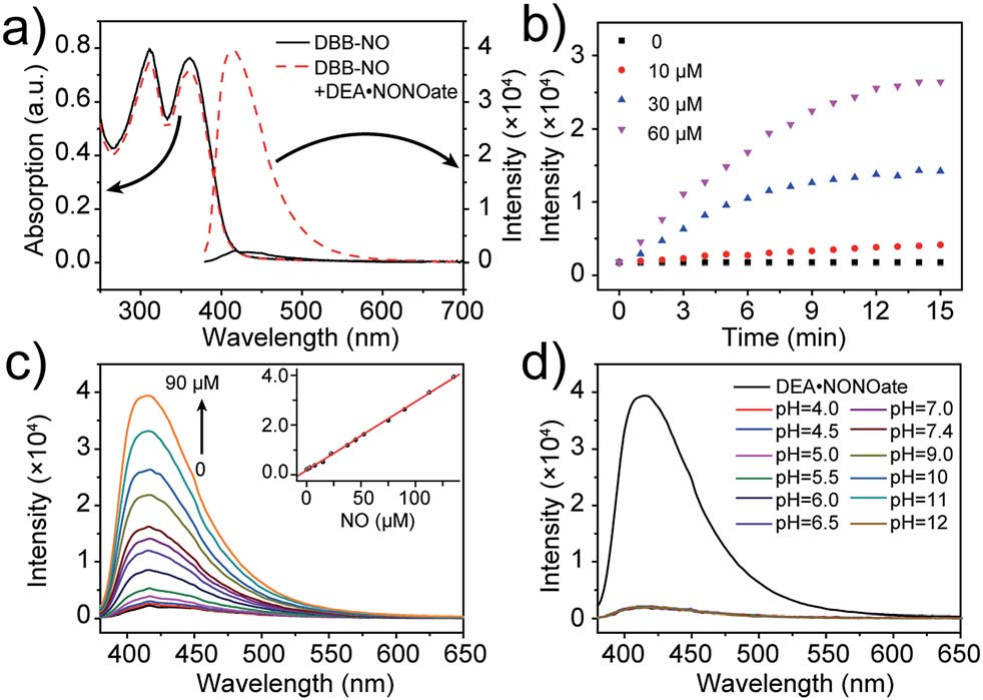
(a) Absorption and fluorescence spectra of **DBB-NO** (5 μM) in the absence and presence of DEA·NONOate. (b) Kinetic profiles of **DBB-NO** with varying DEA·NONOate concentrations. (c) Fluorescence spectra of **DBB-NO** upon the addition of DEA·NONOate (0–90 μM) over a period of 15 min. Inset: the fluorescence intensity at 415 nm against the NO concentration (0–135 μM) is plotted. (d) pH-Dependent fluorescence spectra of **DBB-NO**. The fluorescence spectrum upon the addition of DEA·NONOate (90 μM) over a period of 15 min was used as a reference.

The photophysical properties of **DBB-NO** were studied in pH 7.4 phosphate buffer saline (PBS) solution (without other specifications, all the spectra were recorded in this solution). As expected, **DBB-NO** displays a broadband one-photon absorption spectrum with an excellent molar extinction coefficient (*ε* = 1.3 × 10^5^ M^–1^ cm^–1^) at 361 nm (Fig. S10†) and a very weak fluorescence signal centred at 415 nm with an *Φ*_F_ of 0.17%. We ascribe this low *Φ*_F_ to the intramolecular PeT from the HOMO of OPD to the HOMO of **DBB** (Scheme 1), which significantly quenches the excited singlet exciton of **DBB** and thus results in a low *Φ*_F_. Upon addition of NO (DEA·NONOate was used as the source of NO), the absorption maximum remained almost unchanged while the *Φ*_F_ exhibits an 18-fold enhancement from 0.17% to 9.3% at 415 nm (Fig. 1a). This is because, upon reacting with NO, OPD could be transformed into the benzotriazole unit whose HOMO (–6.73 eV) is considerably close to the HOMO of **DBB**, which is unfavorable for efficient intramolecular PeT. Meanwhile, the as-produced benzotriazole is a leaving group,^16^ which disables intramolecular PeT owing to the increased distance with **DBB**. These results facilitate the competitive radiative transition from the excited singlet state of **DBB** into the ground state for fluorescence emission. Fig. 1b presents the time-dependent fluorescence changes of **DBB-NO** toward NO. The kinetics profiles indicate a high reaction efficiency of **DBB-NO** with NO.

Next, we performed a fluorescence titration experiment of **DBB-NO** toward NO. The gradual fluorescence intensity enhancement of **DBB-NO** at 415 nm exhibits an excellent linear relationship (*R*^2^ = 0.998) with the concentration of DEA·NONOate (Fig. 1c). Based on this, the detection limit (S/N = 3) of **DBB-NO** was determined to be 14 nM, suggesting an excellent sensitivity for fluorescence imaging or activatable PDT.

### High selectivity of **DBB-NO** toward NO

**Fig. 2:**
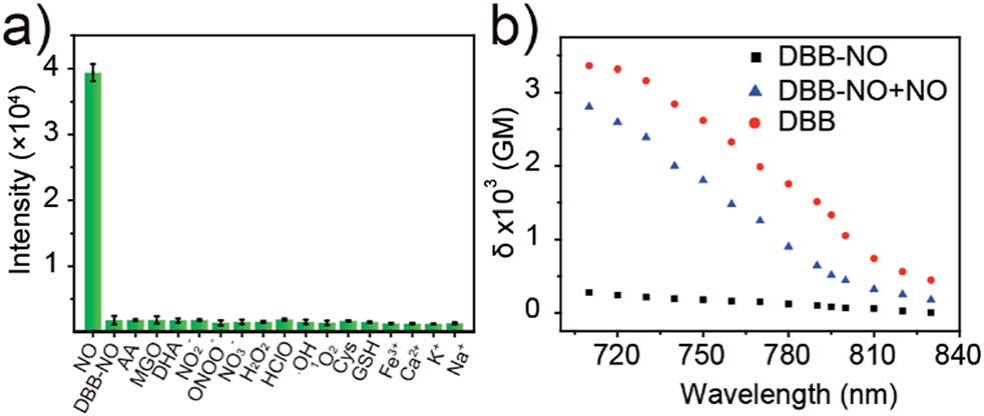
(a) Fluorescence intensity at 415 nm of **DBB-NO** (5 μM) in the presence of various competitive species after 15 min. (b) Two-photon absorption spectra of **DBB-NO** in the absence and presence of DEA·NONOate in pH = 7.4 PBS solution. **DBB** in pH = 7.4 PBS solution was used as a control.

Given the pH variation and existence of other competitive reactive oxygen/nitrogen species (ROS/RNS) in complicated inflammatory conditions, the high selectivity of **DBB-NO** toward NO is of particular importance to avoid the “false positive” fluorescence signals or unexpected phototoxicity toward healthy cells. The negligible fluorescence of **DBB-NO** in a wide pH range demonstrates its excellent pH-stability (Fig. 1d). Furthermore, to verify the selectivity of **DBB-NO**, we screened a wide array of possible competitive reactive oxygen species (H_2_O_2_, HClO, ˙OH, and ^1^O_2_), nitrogen species (NO_2_^–^, ONOO^–^, and NO_3_^–^), biological reductants (cysteine (Cys) and glutathione (GSH)), metal ions (Na^+^, Ca^2+^, K^+^, and Fe^3+^) and other analytes (AA, DHA, and MGO). As anticipated, all these competitive species do not lead to any obvious fluorescence enhancement (Fig. 2a and S11†), suggesting an outstanding selectivity of **DBB-NO** toward NO. From these results, it is reasonable to expect that **DBB-NO** can work in approximate physiological conditions with negligible background fluorescence interference or phototoxicity.

To verify the mechanism for the high selectivity of **DBB-NO**, we analyzed the reaction of **DBB-NO** to NO in PBS solution. The HRMS clearly showed that OPD converted into a cyclized benzotriazole unit with a mass peak of *m*/*z* 119.13 ([M + H]^+^) (Fig. S12†), which is in agreement with previous reports.^16^,^17^ Meanwhile, the occurrence of the characteristic mass peak of **DBB** ([M – 2CH_3_]^+^*m*/*z* 624.88) in HRMS indicates the successful release of the fluorescent and phototoxic **DBB** from **DBB-NO**.

### Two-photon absorption cross-section spectra

*δ* is a key parameter to evaluate the TP-imaging quality and TP-PDT efficiency. To be specific, larger *δ* × *Φ*_F_ and *δ* × *Φ*_Δ_ indicate a better TP-imaging quality and TP-PDT efficiency. Thus, we quantify the *δ* of **DBB-NO** in the absence and presence of NO by a typical Z-scan technique (Fig. 2b).^18^ As anticipated, pure **DBB-NO** possesses a maximum *δ* of 270 GM at 710 nm. Although such a value is comparable to the TP-imaging contrast in other reports,^19^ the ultralow *Φ*_F_ makes **DBB-NO** unusable for TP-imaging due to its low brightness (*δ* × *Φ*_F_). In sharp contrast, upon responding to NO, an extremely elevated *δ* (2800 GM at 710 nm) was observed, which nearly reached the maximum *δ* of **DBB** (3300 GM at 710 nm). This remarkably elevated *δ* of **DBB** in comparison with that of **DBB-NO** should be attributed to the formation of the zwitterionic structure of **DBB**. The zwitterionic structure in a strong polar solvent can provide an additional electric field to energetically increase its *δ*.^13^ Such a remarkably elevated *δ*, accompanying the intrinsic switch on *Φ*_F_ and *Φ*_Δ_, affords **DBB-NO** enormous potential for effective NO-activatable TP-imaging and TP-PDT purposes.

### TP-imaging of exogenous and endogenous NO in living cells

Prior to bioapplications, the low cytotoxicity of **DBB-NO** and excellent biocompatibility of **DBB-NO** were initially verified as shown in Fig. S13 and 14.† For examining the response of **DBB-NO** to NO in the cellular environment, TP-imaging of exogenous NO in HeLa cells was performed (Fig. S15†). In the control group, no discernible fluorescence in the absence of DEA·NONOate demonstrates a negligible background fluorescence of **DBB-NO**. This phenomenon is attributed to the low brightness (*δ* × *Φ*_F_) of **DBB-NO**. After adding 10.0 equiv. DEA·NONOate, a strong fluorescence was observed in living cells within approximately 5 min. Such remarkable fluorescence enhancement indicates that **DBB-NO** can detect exogenous NO even in living cells.

**Fig. 3:**
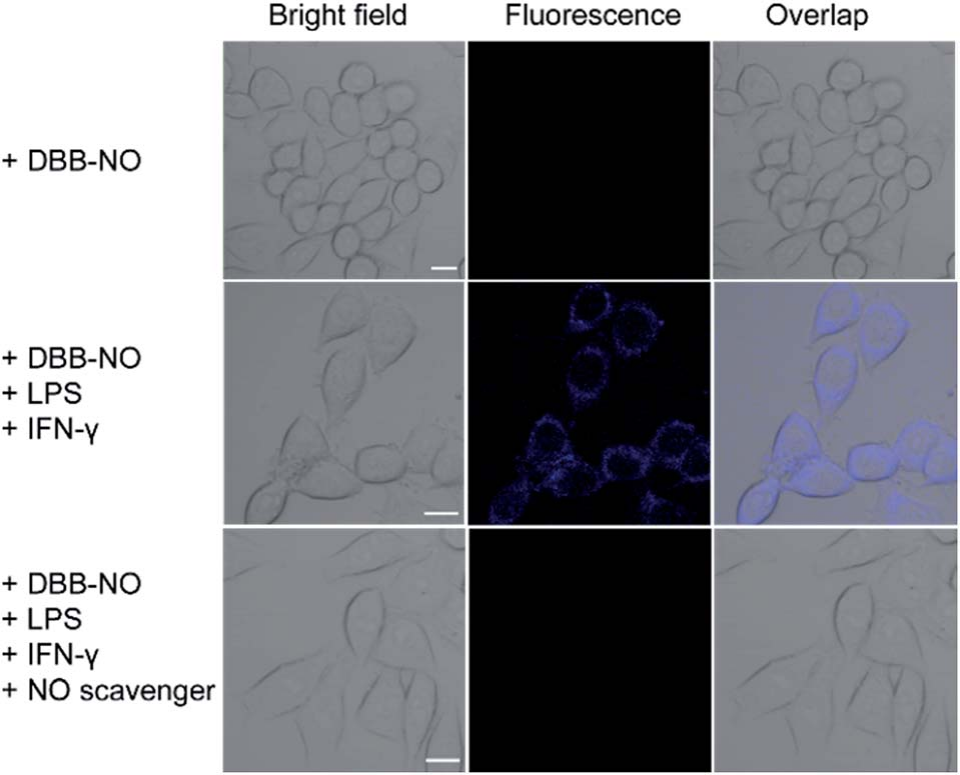
TP-images of RAW 264.7 macrophages stained with **DBB-NO** in the absence or presence of NO stimulants and inhibitor. Fluorescence was recorded at 410–450 nm upon TPE at 750 nm. Scale bar: 10 μm.

After that, we further examine the response of **DBB-NO** to endogenous NO in living cells using the activated RAW 264.7 macrophage. This is because activated macrophages have upregulated endogenous NO (micromolar) in comparison to normal ones. Typically, a normal macrophage could transform into an activated one by the stimulation of lipopolysaccharide (LPS) and interferon-γ (IFN-γ).^20^ Moreover, limiting the proliferation of the activated macrophages is a promising means of controlling inflammation.^21^ As shown in Fig. 3, the normal RAW 264.7 macrophages were incubated with **DBB-NO** and no obvious intracellular fluorescence was detected. In sharp contrast, after the pre-treatment with LPS and IFN-γ, the **DBB-NO** stained RAW 264.7 macrophages (activated macrophages) exhibit a bright intracellular fluorescence. These results clearly show that **DBB-NO** is also capable of detecting endogenous NO in living cells. Remarkably enhanced fluorescence within activated macrophages in comparison with that of the normal macrophages should be attributed to the enhanced TP-fluorescence brightness (*δ* × *Φ*_F_) that is induced by the simultaneous enhancement of *Φ*_F_ and *δ*. More importantly, the different TP-fluorescence brightness in the abovementioned two kinds of macrophage makes **DBB-NO** a potential smart probe to distinguish activated macrophages from normal ones, which is of significant importance in the diagnosis and treatment of human diseases with an inflammatory etiology. To highlight the important role of NO in distinguishing activated macrophages from normal ones, the activated RAW 264.7 macrophages were treated with a powerful NO scavenger (*N*-acetylcysteine, NAC) to eliminate the concentration of endogenous NO. In this context, the intracellular fluorescence of the **DBB-NO** stained RAW 264.7 macrophage was heavily suppressed. From these results, it is evident that **DBB-NO** is endogenous NO-activatable and can serve as a smart probe to distinguish activated macrophages from normal ones.

**Fig. 4:**
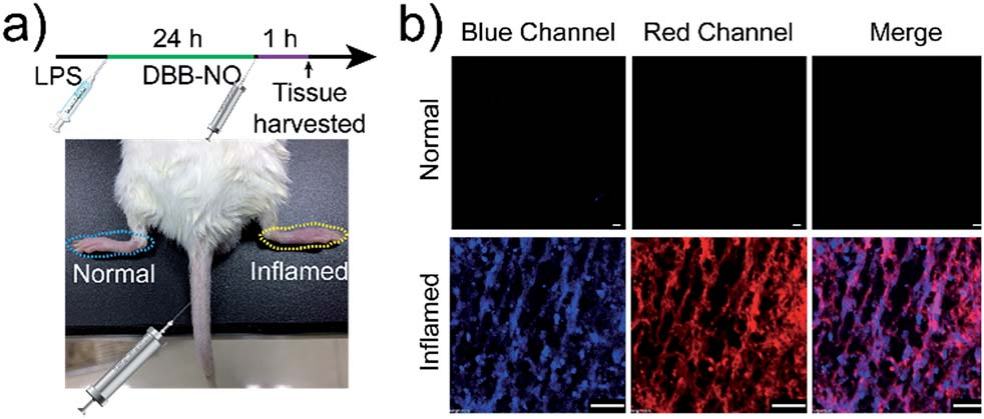
(a) Illustration of the construction of the inflamed mouse model. (b) TP-images of **DBB-NO** in the normal and inflamed tissues. Blue channel was collected at 410–450 nm upon TPE at 750 nm. Red channel was collected at 570–650 nm upon one-photon excitation at 559 nm. Scale bar: 60 μm.

Encouraged by the potential *in vitro* distinguishing ability of **DBB-NO** toward activated and normal macrophages, *in vivo* TP-imaging of NO using **DBB-NO** was then tested in an inflamed mouse model. Using a typical protocol (details in ESI†), we obtained an inflamed mouse model (Fig. 4a) in which inflammatory lumps (yellow circle) in the right rear paw were observed clearly in comparison with the control group (left rear paw, blue circle). Then, **DBB-NO** (150 μL, 10 μM) was intravenously injected into the mouse and the mouse was anesthetized after 1 h. The left and right rear paws were sectioned in different glass slides for subsequent TP-imaging. Pleasingly, the blue channel in Fig. 4b exhibits a distinct fluorescence enhancement in inflamed tissues in comparison with that of normal tissues. Meanwhile, immunostaining of histological sections was performed using macrophage marker CD11b to stain the activated macrophage, in which the activated macrophage can transform nonfluorescent CC11b into a red-emissive dye.^22^ The strong red fluorescence in the red channel suggests the generation of activated macrophages only in the inflamed paw. Moreover, the signals from **DBB-NO** and CD11b are largely overlapped (merge channel) in inflamed tissues. These results indicate not only the specific *in vivo* TP-imaging capability of **DBB-NO** toward endogenous NO but also the *in vivo* distinguishing ability between activated and normal macrophages.

### TP-PDT of activated RAW 264.7 macrophages

**Fig. 5:**
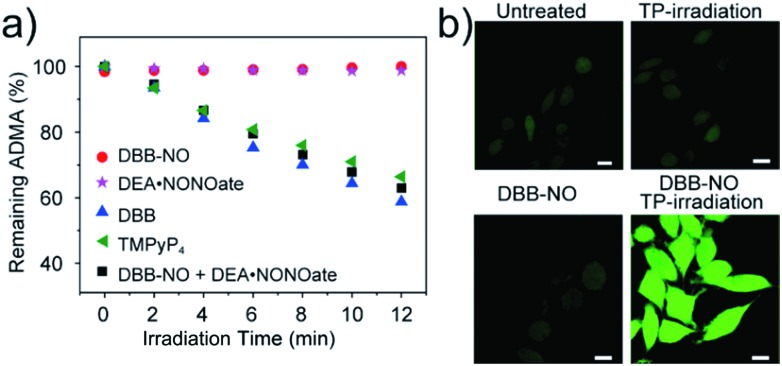
(a) Percentage of unreacted ^1^O_2_ chemical trap (ADMA) in the presence of different samples. 5,10,15,20-Tetrakis(1-methyl-4-pyridinio)porphyrin tetra(*p*-toluenesulfonate) (TMPyP_4_) is a frequently-used commercial PS with an ^1^O_2_ quantum yield of 74%. (b) Detection of intracellular ^1^O_2_ generation using a ROS probe (scale bar: 10 μm), the nonfluorescent ROS probe could generate green-fluorescence in the presence of ROS.

As a proof-of-concept for NO-activatable TP-PDT using **DBB-NO**, we conducted a set of experiments which involved the ROS generation by the well-established photodegradation of anthracene-9,10-diyl-bis-methylmalonate (ADMA) in the presence of **DBB-NO** and **DBB** (Fig. 5a).^23^**DBB** exhibits an exceptional large *Φ*_Δ_ (89%) which is superior to that of commercial TMPyP_4_. The negligible ADMA consumption of **DBB-NO** demonstrates a completely quenched ^1^O_2_ generation (*Φ*_Δ_ = 1.2%). Upon addition of excess DEA·NONOate into the mixture of **DBB-NO** and ADMA solution, the ADMA consumption is comparable to that of **DBB**. After the exclusion of DEA·NONOate interference in ADMA consumption, it is reasonably concluded that the *Φ*_Δ_ of **DBB-NO** can recover to 82%. Such simultaneous enhancement of *δ* and *Φ*_Δ_ allows an unprecedented large TP-PDT efficiency (*δ* × *Φ*_Δ_ = 2296 GM), which precedes the value for the commercial TP-PS by two orders of magnitude.^24^

For proof-of-concept applications, we further checked NO-activatable ^1^O_2_ generation in the activated RAW 264.7 macrophage by fluorescence imaging of a ROS tracker and **DBB-NO** co-stained macrophage. 2,7-Dichlorofluorescein diacetate (DCFH-DA), as a frequently-used ROS tracker, is nonfluorescent and can be oxidized by ROS into a green-emissive DCF. The weak DCF fluorescence within the activated macrophage of the untreated group should be attributed to the endogenous H_2_O_2_, because H_2_O_2_ is also overexpressed in the activated macrophage.^21^ When the activated macrophage was treated with **DBB-NO** or TP-irradiation alone, there is no obvious enhancement of DCF fluorescence in comparison with the untreated group. However, after the co-treatment of the activated macrophage with **DBD-NO** and TP-irradiation, a remarkable enhancement of DCF fluorescence was observed, indicating abundant ^1^O_2_ generation. Combining endogenous NO detection of **DBB-NO** in the activated macrophage, we thus concluded that **DBB-NO** has the capability for NO-activatable ROS (mainly ^1^O_2_) generation even in living cells.

**Fig. 6:**
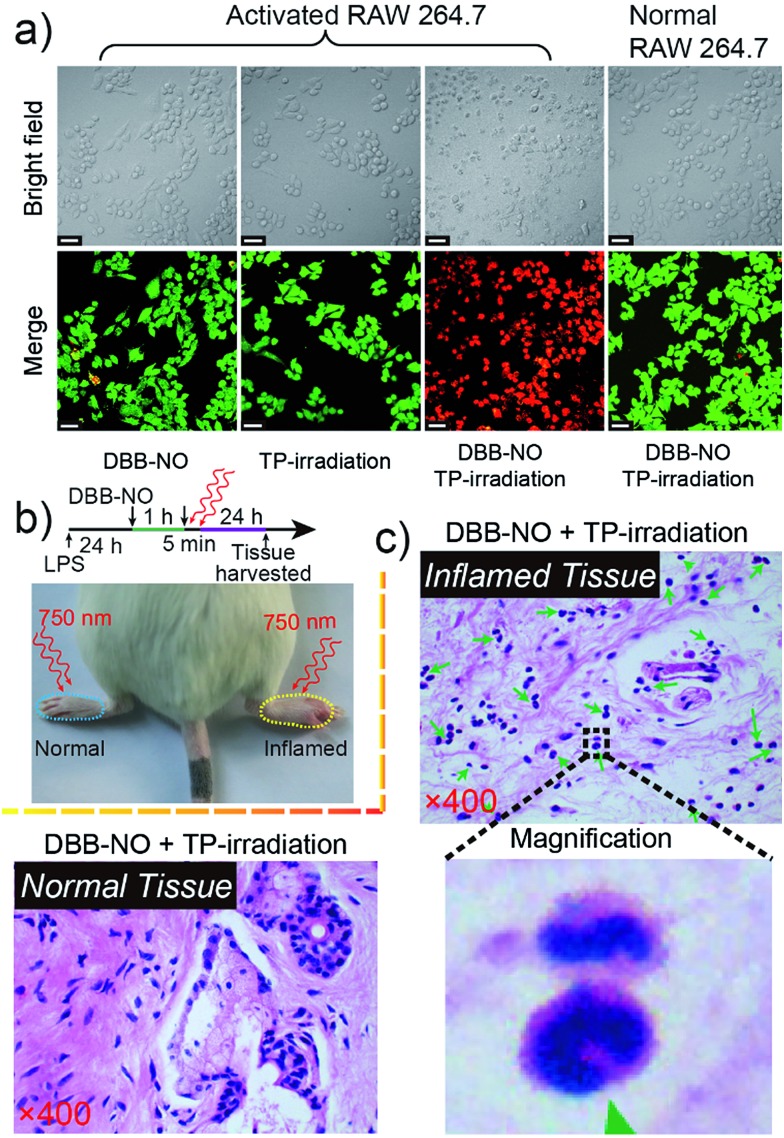
(a) Live/dead assay of the activated RAW 264.7 macrophage after 5 min TP-PDT. The green color represents live cells, and the red color represents dead cells. (b) Illustration for *in vivo* TP-PDT of the inflamed mouse model. (c) Histological H & E assay for the tissues at 24 h after the treatment with **DBB-NO** under TP-irradiation at 750 nm for 5 min. Green arrowheads point to crescent-shaped nuclei and the magnification region gives a representative structure of the crescent-shaped nuclei. The mass emergence of crescent-shaped nuclei represents the macrophage death.

The *in vitro* TP-PDT effect of **DBB-NO** was then evaluated by assessing the cellular phototoxicity. Here, we utilized the calcein AM (living cell) and propidium iodide (PI, dead cell) cellular viability kit to distinguish the dead cells from the living cells (Fig. 6a). Without the cooperation of **DBB-NO** and TP-irradiation, there was no obvious red fluorescence for dead cells in the activated macrophage. This result again proves the low dark cytotoxicity of **DBB-NO** and negligible cytotoxicity caused by TP-irradiation. In the presence of **DBB-NO** and TP-irradiation, a remarkable red fluorescence in the activated macrophages reveals the significant cellular death. Moreover, the obvious cell shrinkage and the formation of numerous blebs of activated macrophage in the bright field of the activated macrophages also indicate the endogenous NO-activatable TP-PDT with high efficiency. Such a highly efficient TP-PDT toward the activated macrophage should be attributed to the simultaneous enhancement of *δ* and *Φ*_Δ_. In addition, we also compared the cellular phototoxicity of **DBB-NO** toward the activated macrophage and normal macrophage. Clearly, **DBB-NO** can only exhibit a highly efficient NO-activatable TP-PDT in the activated macrophage. This is because the normal macrophage is unable to generate sufficient NO to transform the benign **DBB-NO** into phototoxic **DBB**. Furthermore, we performed a quantitative evaluation for the TP-PDT of **DBB-NO** using a MTT (Fig. S16†) assay. The viability of HeLa cells and the normal macrophage incubated with **DBB-NO** remained nearly 100%, while the viability of the activated macrophages incubated with **DBB-NO** was increasingly reduced to 19.1%. In addition, a flow cytometric assay (Fig. S17†) also revealed an efficient TP-PDT only in the activated macrophage. From these results, we demonstrated that **DBB-NO** has an NO-activatable feature for potential TP-PDT in activated macrophages.

With the abovementioned merits, we performed an *in vivo* TP-PDT of **DBB-NO** using the LPS-induced inflamed mouse model. After 5 min TP-irradiation and subsequent housing for another 24 h (Fig. 6b), the skin of the inflamed (right paw) and normal (left paw) tissues were harvested and sectioned for hematoxylin–eosin (H & E) staining assay. Compared to the normal tissue (Fig. 6c) and the control groups treated with **DBB-NO** or TP-irradiation alone (Fig. S18†), the mass emergence of crescent-shaped nuclei (green arrowheads) in inflamed tissue after TP-PDT implied a prominent macrophage apoptosis,^25^ thus illustrating an endogenous NO-activatable phototoxicity of **DBB-NO** to the inflamed tissue. This result preliminarily demonstrated that **DBB-NO** can serve as a smart theranostic agent to selectively destroy activated macrophages and not the normal ones for precision therapy.

## Conclusions

In summary, for the first time we reported a NO-activatable TP-FPS for efficient TP-imaging and TP-PDT in activated macrophages. By coupling NO-responsive OPD with zwitterionic **DBB**, a NO-activatable **DBB-NO** was constructed. Upon responding to NO, **DBB-NO** simultaneously exhibited a remarkably enhanced *Φ*_Δ_ and *Φ*_F_ as well as an extremely NO-elevated *δ*. These properties make **DBB-NO** bright and phototoxic only in the NO-upregulated activated macrophage, thus allowing **DBB-NO** to effectively distinguish activated macrophages from normal ones and subsequently kill them for precision therapy. More importantly, by simply replacing the stimulus-responsive moiety or linker, the zwitterionic structure such as **DBB** with an ultralarge *δ* can act as an active molecular template to construct other pathological condition-activatable materials with the TPA feature for precise theranostics.

## Conflicts of interest

All authors declare no competing financial interest.

## Supplementary Material

Supplementary information
