# Supplementary material for: Nitric oxide activatable photosensitizer accompanying extremely elevated two-photon absorption for efficient fluorescence imaging and photodynamic therapy
Source: Chem Sci. 2017 Nov 27;9(4):999–1005. doi: 10.1039/c7sc04044j (PMC5875019; doi:10.1039/c7sc04044j)
Supplement: Supplementary file 1 [file SC-009-C7SC04044J-s001.pdf]

Electronic Supplementary Material (ESI) for Chemical Science.
This journal is © The Royal Society of Chemistry 2017

Supplementary Information

Nitric oxide activatable photosensitizer accompanying extremely elevated two-photon absorption for efficient fluorescence imaging and photodynamic therapy

Wenbo Hu,[a,b†] Meng Xie,[a] Hui Zhao,[a] Yufu Tang,[a] Song Yao,[c] Tingchao He,[c] Chuanxiang Ye,[c] Qi Wang,[b] Xiaomei Lu,[b] Wei Huang[b,d] and Quli Fan[a]*

[a] *Key Laboratory for Organic Electronics and Information Displays & Institute of Advanced Materials (IAM), Jiangsu National Synergetic Innovation Center for Advanced Materials (SICAM), Nanjing University of Posts & Telecommunications, Nanjing 210023, China. E-mail:* iamqlfan@njupt.edu.cn.

[b] *Key Laboratory of Flexible Electronics (KLOFE) & Institute of Advanced Materials (IAM), Jiangsu National Synergetic Innovation Center for Advanced Materials (SICAM), Nanjing Tech University (NanjingTech), Nanjing 211816, China. E-mail: wei-huang@njtech.edu.cn.*

[c] *Key Laboratory of Optoelectronic Devices and Systems of Ministry of Education and Guangdong Province, College of Physics Science & Technology, Shenzhen University, Shenzhen 518060, China.*

d. Shaanxi Institute of Flexible Electronics (SIFE), Northwestern Polytechnical University (NPU), Xi'an 710072, China.

†Present address: Key Laboratory of Flexible Electronics (KLOFE) & Institute of Advanced Materials (IAM), Jiangsu National Synergetic Innovation Center for Advanced Materials (SICAM), Nanjing Tech University (NanjingTech), Nanjing 211816, China.

Contents

1. Materials and instruments........2
2. Theoretical calculation........3
3. Synthesis and characteristic........3
4. Optimal experiment of **DBB-NO**........5
5. Cytotoxicity and biocompatibility of **DBB-NO**........6
6. Procedure for cell imaging........7
7. TP-PDT........8
8. Supporting figures........10
9. References........17

1. Materials and instruments

Materials: 2, 2-((2, 5-diiodo-1, 4-phenylene) bis (oxy)) bis (N, N-diethylethanamine) (monomer 2) was reported in our previous literature,[1] and used as received. Unless otherwise noted, other reagents were purchased from Sigma Aldrich and used without additional purification. All solvents were purchased from either Fisher Scientific or Aldrich and used as received. Dulbecco's modified Eagle's medium (DMEM, Gibco, America) was purchased from Gene Tech Co. Lipopolysaccharide (LPS), interferon-γ (IFN-γ) and N-acetyl-L-cysteine (NAC) was purchased from Abcam or Sigma-Aldrich.

Instruments: All the calculations were done with Gaussian09 program (Revision B.01). NMR spectra were recorded on a Bruker Ultra Shield Plus 400 MHz spectrometer (^{1}H: 400 MHz, ^{13}C: 100 MHz) and referenced to tetramethylsilane (TMS) as the internal standard, the following abbreviations are used to explain the multiplicities: s=singlet, d=doublet, t=triplet, q=quartet, m=multiplet. Mass spectra were obtained on a matrix-assisted laser desorption/ionization time of flight mass spectrometry (MALDI-TOF MS, Bruker AutoFlex III system). The steady-state absorption data and photoluminescence spectra were measured by a Shimadzu UV-3600 Plus ultraviolet-visible-near-infrared spectrophotometer and an Edinburgh FLSP920 spectrofluorometer, respectively. The absolute fluorescence quantum yield measured using an Edinburgh FLSP920 fluorescence spectrophotometer equipped with an integrating sphere and a xenon lamp. Two-photon absorption cross-sections were measured by the Z-scan technique using home-built experimental setup.[2] The methyl thiazolyl tetrazolium (MTT) assay was performed by a PowerWave XS/XS2 microplate spectrophotometer (BioTek, Winooski, VT). The laser source is a Ti:sapphire system that produced 100 fs pulses at a repetition of 80 MHz. All images were acquired on Leica TCS SP5X Confocal Microscope System equipped with Leica HCX PL APO 63x/1.20 W CORR CS, 405 nm Diode laser, white laser (470 nm to 670 nm, with 1 nm increments, with 8 channels AOTF for simultaneous control of 8

laser lines, each excitation wavelength provides 1.5 mV), and Ti-Sapphire laser (~4 W at 800 nm) which corresponded to approximately 1% (~40 mW at 800 nm) average power in the focal plane.

Statistical analysis: The statistical analysis of the samples was undertaken using a Student's t-test, and *p*-values < 0.05 were considered statistically significant. All data reported are means ± standard deviations, unless otherwise noted.

2. Theoretical calculation

The corresponding excitation energy and molecular orbitals of DBB and OPD were calculated using Density Functional Theory (DFT). All the calculations are completed in Gaussian 09.

3. Synthesis and characteristic

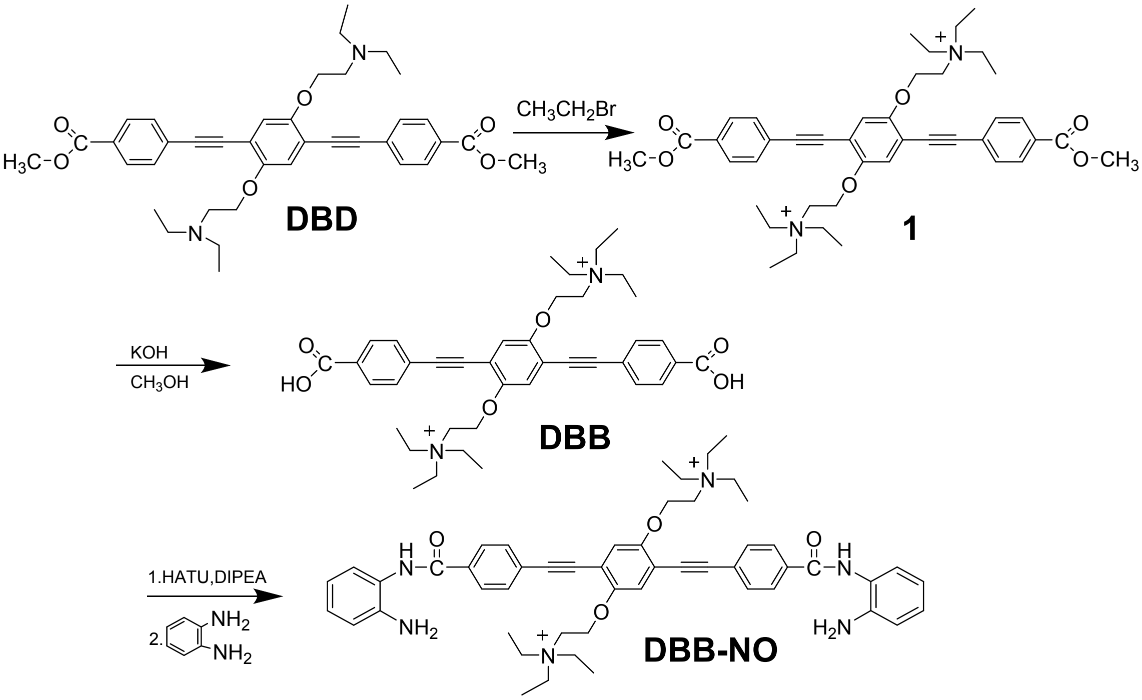

Scheme S1 Synthetic route of **DBB-NO**.

2,2'-((2,5-bis((4-(methoxycarbonyl)phenyl)ethynyl)-1,4-phenylene)bis(oxy))bis(N,N,N-triethylethan-1-aminium) bromide (1)

DBD (0.5 g, 0.8 mmol) were dissolved in 10 mL of Tetrahydrofuran (THF) and 15 mL Bromoethane was added. The reaction mixture was stirred at reflux for 48 h. The mixture was filtered, the solid was separated and washed with CH_2Cl_2 three times to

give 0.46g emerald green powders (yield 92%). ^{1}H NMR (400 MHz, Methanol-d_4) δ 8.03 (d, J = 8.4 Hz, 4H), 7.57 (d, J = 8.4 Hz, 4H), 7.04 (s, 2H), 4.17 (t, J = 5.5 Hz, 4H), 3.95 – 3.91 (m, 6H), 3.06 (s, 4H), 2.78 (d, J = 6.4 Hz, 8H), 1.11 (t, J = 7.1 Hz, 12H). ^{13}C NMR (100 MHz, MeOD) δ 166.27, 152.72, 131.26, 131.26, 131.25, 131.22, 130.05, 129.39, 129.38, 129.38, 127.32, 117.17, 113.86, 113.68, 94.18, 94.06, 87.66, 87.58, 64.31, 62.63, 55.56, 53.53, 53.53, 51.48, 51.48, 50.95, 48.79, 8.16, 6.65. MALDI-TOF MS (m/z): calcd for $C_{42}H_{54}N_2O_6$, exact mass: 682.40; found: m/z 653.50 $[M-2CH_3]^+$.

2,2'-((2,5-bis((4-carboxyphenyl)ethynyl)-1,4-phenylene)bis(oxy))bis(N,N,N-triethylethan-1-aminium) bromide (DBB)

The **1**（0.46 g, 0.55 mmol）was dissolved in 10 mL of methanol and potassium hydroxide aqueous solution (2 g in 15 mL water) was stirred at 60 °C for 6 h. After the stirring, the obtained water layer was separated and washed with dichloromethane three times. Diluted hydrochloric acid was added into water layer until the solution is neutral. The solvent was evaporated under reduced pressure. Diluted hydrochloric acid was added into water layer until the green precipitate not occur, the green precipitation was filtered and washed with water twice and brine once and dried over anhydrous sodium sulfate to give 0.4 g green powders (yield 86.9%). ^{1}H NMR (400 MHz, Methanol-d_4) δ 8.06 (d, J = 8.3 Hz, 4H), 7.64 (d, J = 8.2 Hz, 4H), 7.37 (d, J = 13.9 Hz, 2H), 4.59 – 4.45 (m, 4H), 3.90 – 3.67 (m, 4H), 3.47 (dq, J = 34.7, 6.9 Hz, 11H), 1.35 (t, J = 6.8 Hz, 18H). ^{13}C NMR (100 MHz, MeOD) δ 167.49, 152.86, 152.70, 131.19, 131.15, 130.71, 129.62, 129.60, 127.13, 127.11, 117.17, 116.94, 113.88, 113.71, 94.28, 94.16, 87.44, 64.29, 62.69, 55.64, 53.59, 50.91, 48.75, 8.14, 6.68. MALDI-TOF MS (m/z):calcd for $C_{40}H_{50}N_2O_6$, exact mass: 654.37; found: 625.556$[M-2CH_3]^+$.

2,2'-((2,5-bis((4-((2-aminophenyl)carbamoyl)phenyl)ethynyl)-1,4-phenylene)bis(oxy))bis(N,N,N-triethylethan-1-aminium) bromide (DBB-NO)

The **2** (0.15 g, 0.18 mmol) and HATU (0.27 g, 0.71 mmol) was dissolved in 10 mL of dry N,N-Dimethylformamide (DMF), and then DIPEA (0.5 mL) was added. The mixture was allowed to react for 40 min at room temperature, and then DIPEA (0.2 mL) and o-diaminobenzene (77.0 mg, 0.78 mmol) were added. After stirring at 50 °C

for 24 h, the mixture was concentrated under vacuum and the crude product was purified by silica column chromatography (CH_2Cl_2:CH_3OH=5:1) to give 19 mg brown powder (compound **DBB-NO**) (yield 12.6%). ^1H NMR (400 MHz, Methanol-d_4) δ 8.09 – 7.98 (m, 4H), 7.66 (dd, J = 16.5, 8.3 Hz, 4H), 7.30 (d, J = 21.8 Hz, 2H), 7.23 – 7.15 (m, 2H), 7.10 (t, J = 8.5 Hz, 2H), 6.92 (d, J = 11.2 Hz, 2H), 6.78 (t, J = 8.0 Hz, 2H), 4.51 (s, 2H), 4.25 (t, J = 5.1 Hz, 2H), 3.83 (s, 2H), 3.48 (dq, J = 14.6, 7.2 Hz, 8H), 3.11 (t, J = 4.6 Hz, 2H), 2.84 (q, J = 7.1 Hz, 4H), 1.36 – 1.25 (m, 18H). ^{13}C NMR (100 MHz, MeOD) δ 177.78, 171.63, 168.83, 166.44, 153.46, 152.35, 142.49, 134.28, 134.17, 131.26, 131.19, 127.88, 127.82, 127.31, 126.32, 126.29, 126.13, 123.70, 118.25, 118.23, 117.35, 117.31, 116.86, 116.72, 113.82, 113.57, 94.17, 94.00, 87.34, 87.29, 72.46, 65.72, 63.00, 62.52, 58.68, 55.52, 53.46, 50.64, 7.06, 6.62. MALDI-TOF MS (m/z):calcd for $C_{52}H_{62}N_6O_4$, exact mass: 834.48; found: m/z 805.649$[M-2CH_3]^+$.

4. Optimal experiment of DBB-NO

Absolute fluorescence quantum yield. We employed an Edinburgh FLSP920 fluorescence spectrophotometer equipped with an integrating sphere to measure the absolute fluorescence quantum yield according to the following formula:

$$\Phi_F = \frac{E_{Sam}^{Sample\ emission}}{E_{Ref}^{reference\ scatter} - E_{Sam}^{Sample\ Scatter}}$$

The calculated Φ_F of **DBB-NO** and **DBB** in PBS buffer solution is 0.17% and 9.3 %, respectively.

Determination of the detection limit. The detection limit was calculated according to the method reported in the previous literature[3]. The fluorescence emission spectrum of **DBB-NO** was measured by five times and the standard deviation of blank measurement was achieved. The fluorescence intensity at 415 nm was plotted as a concentration of NO. The detection limit was calculated with the following equation:

$$\text{Detection limit} = 3\sigma/k$$

Where σ is the standard deviation of blank measurement, k is the slope between the fluorescence intensity versus NO concentration.

Two-photon absorption cross-sections (δ) spectra. The δ spectrum of target molecule was acquired via a point-to-point method. Briefly, the δ of different sample in PBS at different TP-excitation wavelength (710, 720, 730, 740, 750, 760, 770, 780, 790, 800, 810, 820, 830) were tested, respectively. Then the δ against different TPE wavelength were ploted as the two-photon absorption cross-sections spectra (Fig. 2b).

5. Dark cytotoxicity and biocompatibility of DBB-NO

Cell culture. HeLa cells and Murine RAW 264.7 macrophages lines were incubated respectively in 75-cm^2 flasks containing DMEM supplemented with 10% fetal bovine serum (FBS), 1% penicillin streptomycin glutamine (GIBCO) at 37ºC in a humidified incubator of 5% CO_2. Then, cells were carefully harvested and split when they reached a certain confluence for following use.

Dark cytotoxicity. The MTT assay was used to determine the dark cytotoxicity of **DBB-NO** in HeLa cells and RAW 264.7 macrophages according to manufacturer's guidelines. The cells were further inculcated in medium containing different doses of **DBB-NO** for 24 h. Then, 10 μL MTT (0.5 mg/mL) solution was added into each well. After 3 h incubation at 37 °C, the supernatant was removed and 200 μL of dimethyl sulfoxide (DMSO) was added. A PowerWave XS/XS2 microplate spectrophotometer was used to record the absorbance intensity at 490 nm. The cellular viability relative to the untreated cells (control group) was calculated following equation:

$$\frac{A_{\text{sample}}}{A_{\text{control}}}$$

in which A_{sample} and A_{control}, respectively, represent the average absorption of groups containing **DBB-NO** and untreated cells.

6. TP-imaging of NO in living cells

TP-imaging of exogenous NO in HeLa cells. HeLa cells were seeded in glass-bottom dishes and grown till 70 ~ 80% confluences. Subsequently, cells were

incubated with **DBB-NO** (2 μM prepared in fresh media from a buffer stock) in the absence and presence of DEA·NONOate for 6 h. Cells were then washed three times with PBS, then imaged with the Leica TCS SP5X Confocal Microscope System. Images of **DBB-NO** stained cells were collected at 410 ~ 450 nm by using a Ti-Sapphire laser excitation wavelength at 750 nm.

TP-imaging of endogenous NO in RAW 264.7 macrophage. The cell experiment can be divided into three groups. The first group is that RAW 264.7 macrophages were incubated with 2 μM **DBB-NO** for 6 h. Then macrophages were washed by PBS buffer before imaging. In the second group, RAW 264.7 macrophages were incubated with 2 μM **DBB-NO** for 6 h, washed by PBS buffer and subsequently incubated with Lipopolysaccharide (LPS, 1 μg/mL) and interferon-γ (IFN-γ, 1 μg/mL) for 1 h prior to imaging. In the third group RAW 264.7 macrophage cells were incubated with 2 μM **DBB-NO** for 6 h, washed by PBS buffer and subsequently incubated with Lipopolysaccharide (LPS, 1 μg/mL), interferon-γ (IFN-γ, 1 μg/mL) and N-acetylcysteine (NAC, 2 mM) respectively for 1 h prior to imaging.

Animal model and LPS-induced tissue inflammation. All animal studies were performed in strict compliance with the National Institutes of Health (NIH) guidelines for the care and use of laboratory animals (NIH Publication no. 85-23 Rev. 1985) and approved by the Animal Ethics Committee of Simcere Pharmaceutical Group (Nanjing, China). Three mice (6 weeks of age) were purchased from Shanghai Laboratory Animal Center, Chinese Academy of Sciences (SLACCAS) and fed in an axenic environment. Briefly, 200 μL LPS (1 mg/mL) was subcutaneously injected into the right rear paw of mice to obtain inflammatory lumps. The left rear paw without any treatment serve as control. After 24 h, **DBB-NO** (150 μL, 10 μM) was intravenously injected into mouse, the mouse was anesthetized after 1 h. After 1 h, the skin of the inflamed (right paw) and normal (left paw) tissues were harvested and embedded in tissue-freezing medium frozen and then the left and right rear paws were sectioned into 300 μm segments in different glass slides.

TP-imaging of NO in inflamed tissue. The immunostaining and TP-imaging of histological sections were carefully washed with PBS and fixed with 4% paraformaldehyde for 25 min at room temperature. After that, the tissues were treated with 1% bovine serum albumin in PBS for 1 h. Then macrophage marker CD11b were used to stain activated macrophage cells. Leica TCS SPS X microscope was used to take images. For CD11b, the excitation wavelength is 630 nm and the collection wavelength range is from 650-670 nm. For **DBB-NO**, the collection wavelength range is from 410-450 nm upon TP-excitation at 750 nm.

7. TP-PDT

$^1O_2$ quantum yield measurements via the chemical method. Water-soluble ADMA was used as the $^1O_2$-trapping agent, and $TMPyP_4$ was used as the standard photosensitizer. In the experiments, 20 μL of ADMA in PBS solution (1 mg/mL^{-1}) and 10 μL DEA·NONOate (200 equiv) was added to probe (5 μM) in PB buffer solution, and light (365 nm) with a power density of 6.5 mW cm^{-2} was employed as the irradiation source. The absorption of ADMA at 261 nm was recorded at various irradiation times to obtain the decay rate of the photosensitizing process. $TMPyP_4$ in PB buffer solution was used as the reference (r), and Φ_r = 74% in water.[4] The $^1O_2$ quantum yield of the photosensitizer (Φ_Δ) can be calculated according to the following formula:

$$\Phi_\Delta = \Phi_r * \frac{K_{\text{sample}} * A_r}{K_r * A_{sample}}$$

Where K_{sample} and K_r are the decomposition rate constants of ADMA by the **DDB-NO** and $TMPyP_4$, respectively. A_{sample} and A_r represent the light absorbed by the **DDB-NO** and $TMPyP_4$, respectively, which are determined by integration of the optical absorption bands in the wavelength range 230 - 800 nm.

Intracellular $^1O_2$ detection. Raw264.7 macrophages were cultured in confocal microscope dishes to 20~30% confluence in complete DMEM medium and cultured

for 24 h. Then, the medium was aspirated, washed with PBS, treated with medium containing 20 μM **DBB-NO** for 6 h, and then further incubated with 10 μM $^1O_2$ probe DCFH-DA. After incubation for 20 min, the cells were washed again and exposed under two-photon confocal laser at 750 nm for 5 min to generate $^1O_2$. The green fluorescent signals of DCF at 505 nm ~ 555 nm were captured under the irradiation at 488 nm by using laser confocal microscopy.

***in vitro* TP-PDT using confocal fluorescence imaging**. The TP-PDT of macrophages can be divided into four groups. The first group is that RAW 264.7 macrophages were incubated with 2 μM **DBB-NO** for 6 h. Then macrophages were washed by PBS buffer and subsequently incubated with LPS (1 μg/mL) and IFN-γ (1 μg/mL) for 1 h to activate macrophage before testing cellular viability. In the second group, RAW 264.7 macrophage cells were incubated with LPS (1 μg/mL) and IFN-γ (1 μg/mL) for 1 h to activate macrophage. After that, macrophages were exposed to 1 kHz femtosecond laser TP-irradiation at 750 nm with power density of ~1.0 W cm^{-2} for 5 min (without specifically states, all the TP-irradiation in following at 750 is same as this) and then these macrophages were incubated for 4 hours before testing cellular viability. In the third group, RAW 264.7 macrophages were incubated with 2 μM **DBB-NO** for 6 h, washed by PBS buffer and subsequently incubated with LPS (1 μg/mL), IFN-γ (1 μg/mL) and N-acetylcysteine (NAC, 2 mM) respectively for 1 h. After that, macrophages were exposed to TP-irradiation at 750 nm for 5 min and then these macrophages were incubated for 4 hours before testing cellular viability. In the fourth group, normal RAW 264.7 macrophages were incubated with 2 μM **DBB-NO** for 6 h. After that, macrophages were exposed to TP-irradiation at 750 nm for 5 min and then these macrophages were incubated for 4 hours before testing cellular viability. For cellular viability assessment, the macrophages were further incubated 1 h in a medium containing 1 μM of calcein AM and 500 nM of propidium iodide (PI). Calcein AM with green emission represents living cells and PI with red emission represent dead cells. For Calcein AM, the excitation wavelength is 488 nm and the collection wavelength range

is from 510-530 nm. For PI, the collection wavelength range is from 610-630 nm upon excitation at 559 nm.

***in vitro* TP-PDT using MTT assay.** For evaluating the NO-activatable TP-PDT of **DBB-NO** in activated macrophage, RAW 264.7 macrophages were incubated with **DBB-NO** at different concentration for 6 h, washed by PBS buffer and subsequently incubated with LPS (1 μg/mL) and IFN-γ (1 μg/mL). After 1 h, these prepared macrophages were exposed to the TP-irradiation at 750 nm for 5 min. After incubating another 12 h, the cell viability of macrophages was measured by typical MTT assay. HeLa cells and normal RAW 264.7 macrophage (without treatment of LPS and IFN-γ) under the same experimental conditions were also performed for direct comparison.

***in vitro* TP-PDT using flow cytometric assay**. To get a quantitative evaluation of TP-PDT effect of **DBB-NO**, the same four groups as above mentioned for were prepared in 4 well plates. The first group is that RAW 264.7 macrophages were incubated with 2 μM **DBB-NO** for 6 h. Then macrophages were washed by PBS buffer and subsequently incubated with LPS (1 μg/mL) and IFN-γ (1 μg/mL) for 1 h to activate macrophage before testing cellular viability. In the second group, RAW 264.7 macrophage cells were incubated with Lipopolysaccharide (LPS, 1 μg/mL) and interferon-γ (IFN-γ, 1 μg/mL) for 1 h to activate macrophage. After that, macrophages were exposed to TP-irradiation at 750 nm for 5 min and then these macrophages were incubated for 4 hours before testing cellular viability. In the third group, RAW 264.7 macrophages were incubated with 2 μM **DBB-NO** for 6 h, washed by PBS buffer and subsequently incubated with LPS (1 μg/mL), IFN-γ (1 μg/mL) and N-acetylcysteine (NAC, 2 mM) respectively for 1 h. After that, macrophages were exposed to TP-irradiation at 750 nm for 5 min and then these macrophages were incubated for 4 hours before testing cellular viability. In the fourth group, RAW 264.7 macrophages were incubated with 2 μM **DBB-NO** for 6 h. After that, macrophages were exposed to TP-irradiation at 750 nm for 5 min and then these macrophages were incubated for 4 hours before testing cellular viability. For cellular viability assessment, the collected

RAW 264.7 macrophages were centrifuged and washed by PBS twice before staining by Annexin FITC/PI cell apoptosis kit according to the product's protocol. Finally, flow cytometer analysis was performed by FlowSight Imaging Flow Cytometer.

***in vivo* TP-PDT**. The LPS-induced mouse model used for *in vivo* TP-PDT was same as the model for in vivo TP-imaging of endogenous NO. After acquiring the inflamed model, **DBB-NO** (150 μL, 10 μM) was intravenously injected into mouse. 1 h later, the inflamed (right paw) and normal (left paw) region of mouse was exposed to TP-irradiation at 750 nm for 5 min. After housing for another 24 h, the skin of the inflamed (right paw) and normal (left paw) tissues were harvested and sectioned for hematoxylin-eosin (H&E) staining assay. The inflamed tissues treated with DBB-NO or TP-irradiation alone were also examined to exclude the injury toward inflamed tissues from **DBB-NO** and TP-irradiation alone.

8. Supporting figures

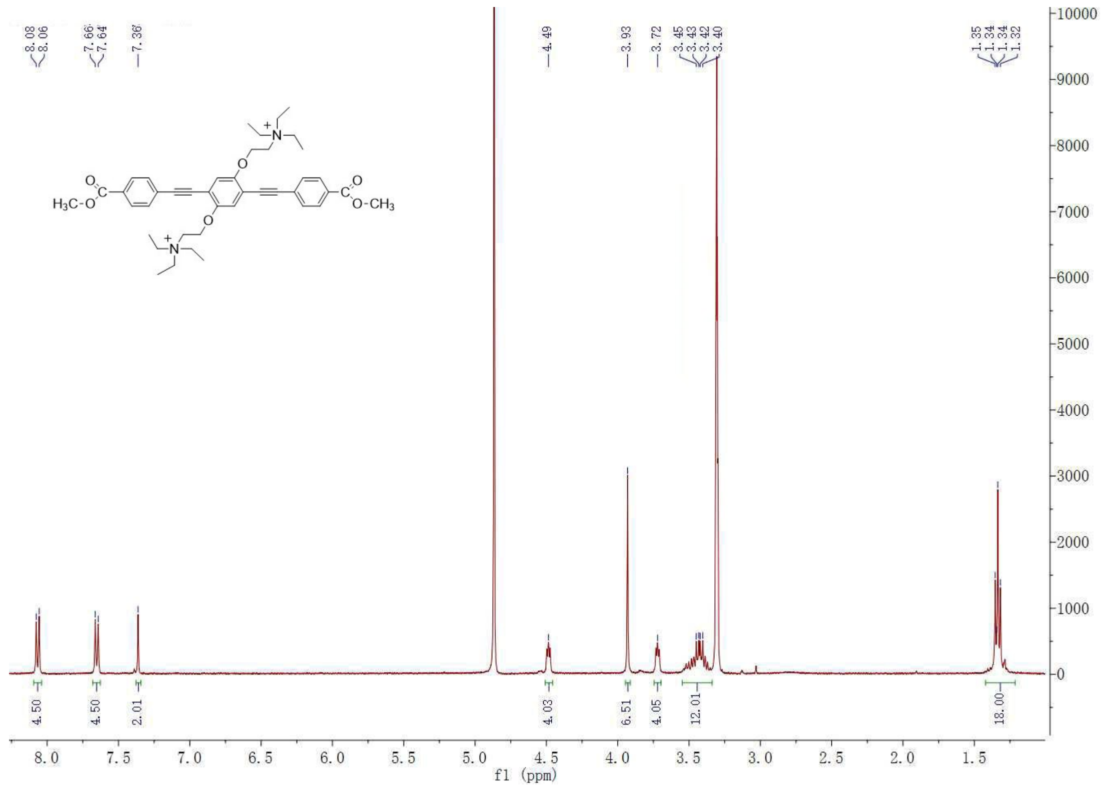

Fig. S1. ^{1}H NMR spectrum (400 MHz, 298 K) of Compound 1 in Methanol-d_4 with concentration of 2 mM.

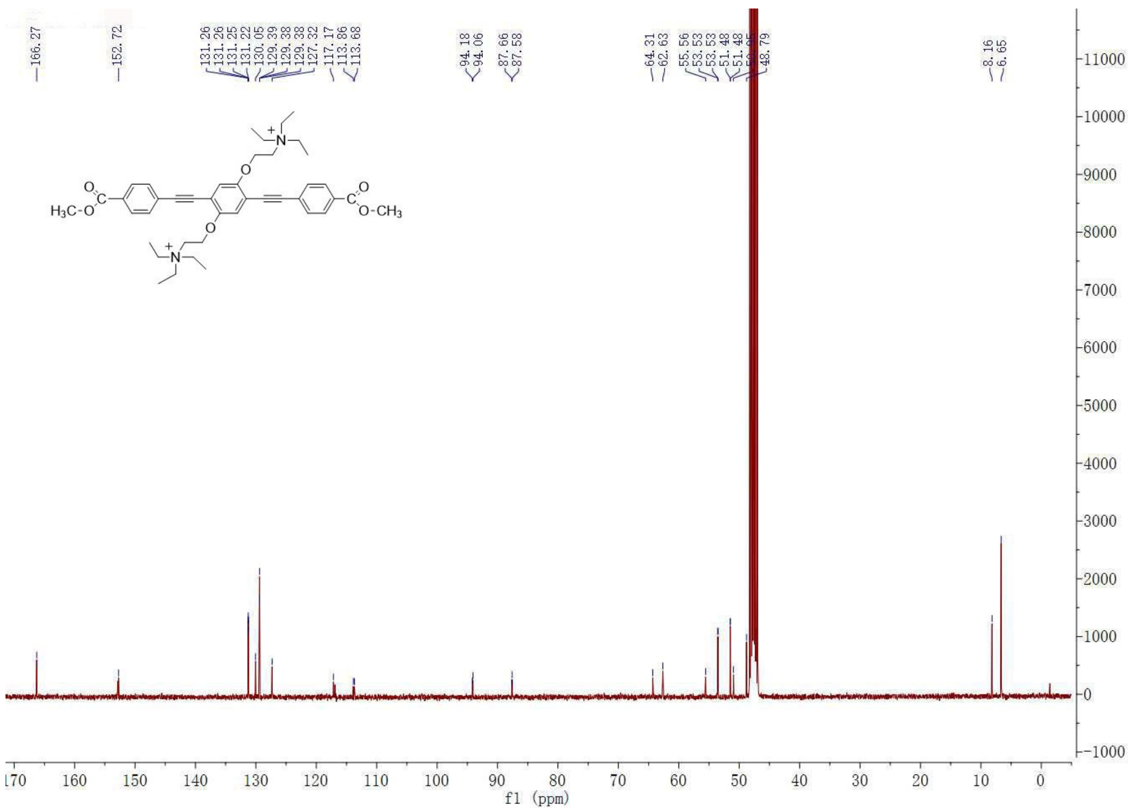

Fig. S2. ^{13}C NMR spectrum (100 MHz, 298 K) of Compound 1 in Methanol-d_4 with concentration of 2 mM.

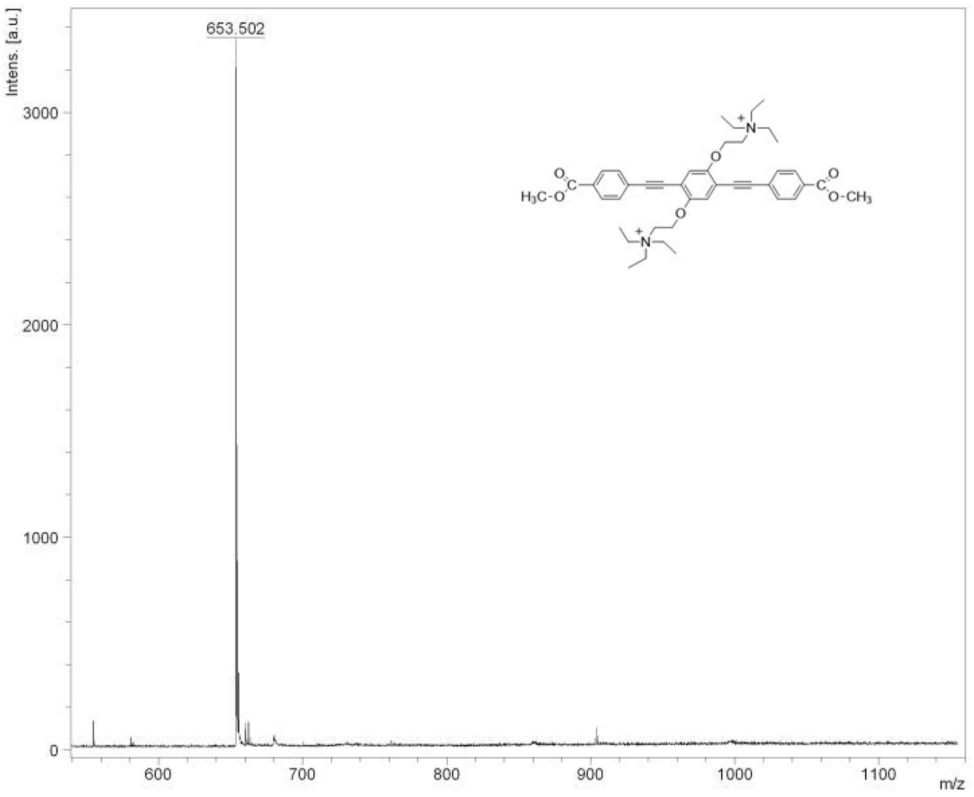

Fig. S3. MALDI-TOF MS chart of Compound 1.

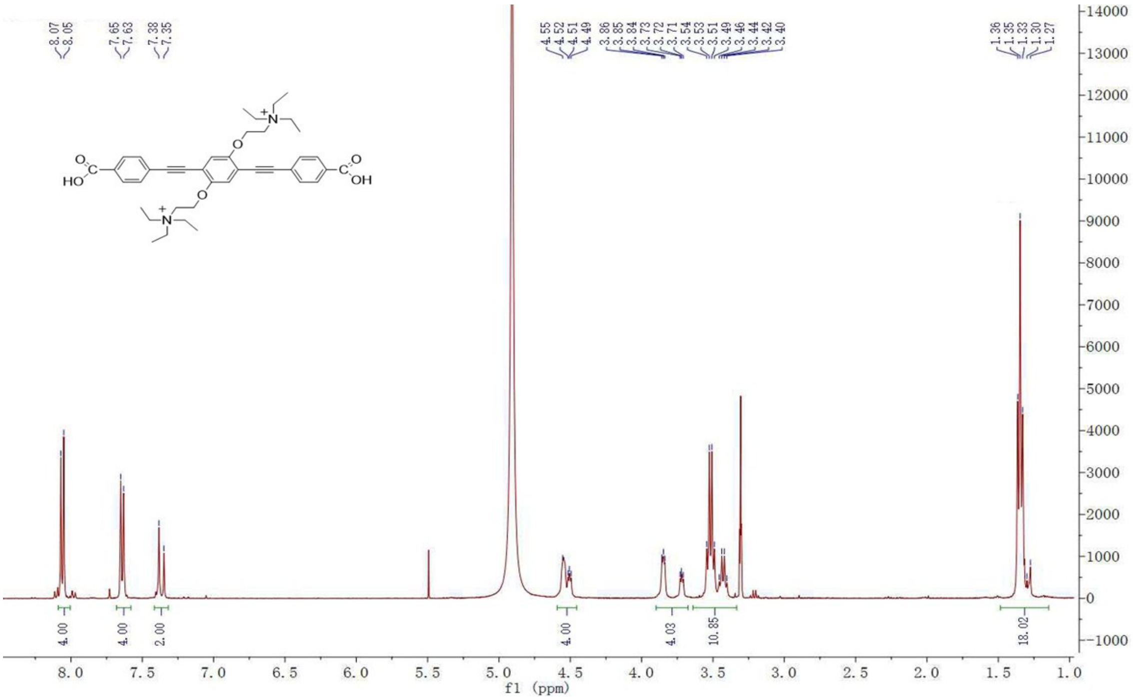

Fig. S4. ^{1}H NMR spectrum (400 MHz, 298 K) of Compound **DBB** in Methanol-d_4 with concentration of 2 mM.

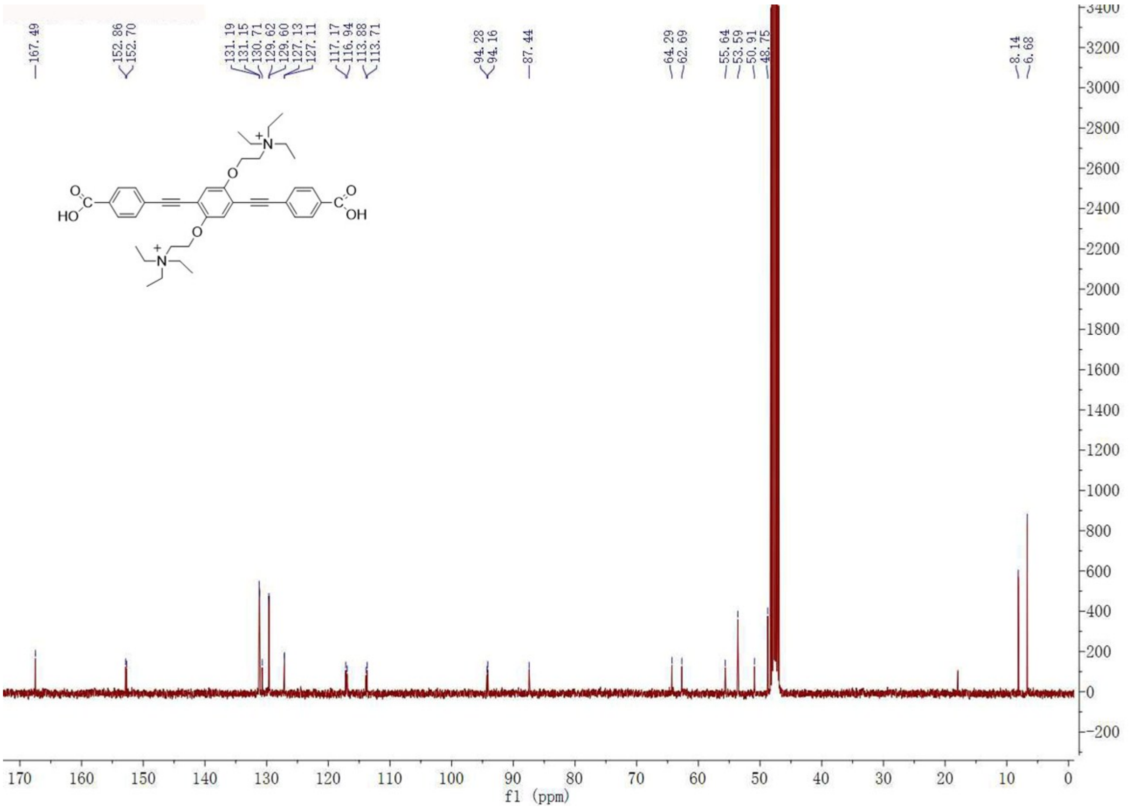

Fig. S5. ^{13}C NMR spectrum (100 MHz, 298 K) of Compound **DBB** in Methanol-d_4 with concentration of 2 mM.

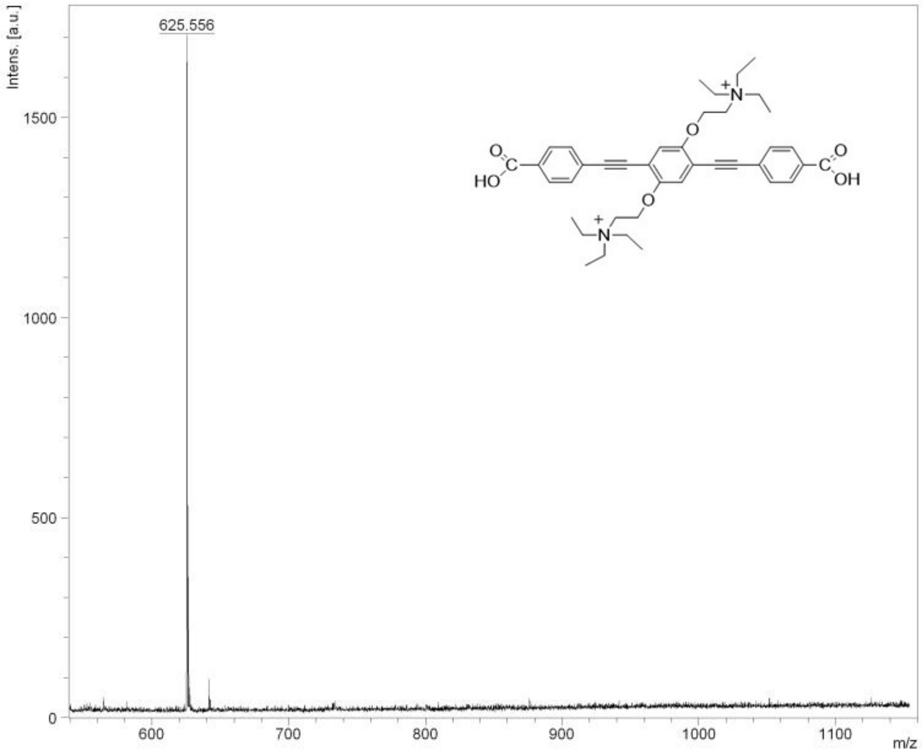

Fig. S6. MALDI-TOF MS of Compound **DBB**.

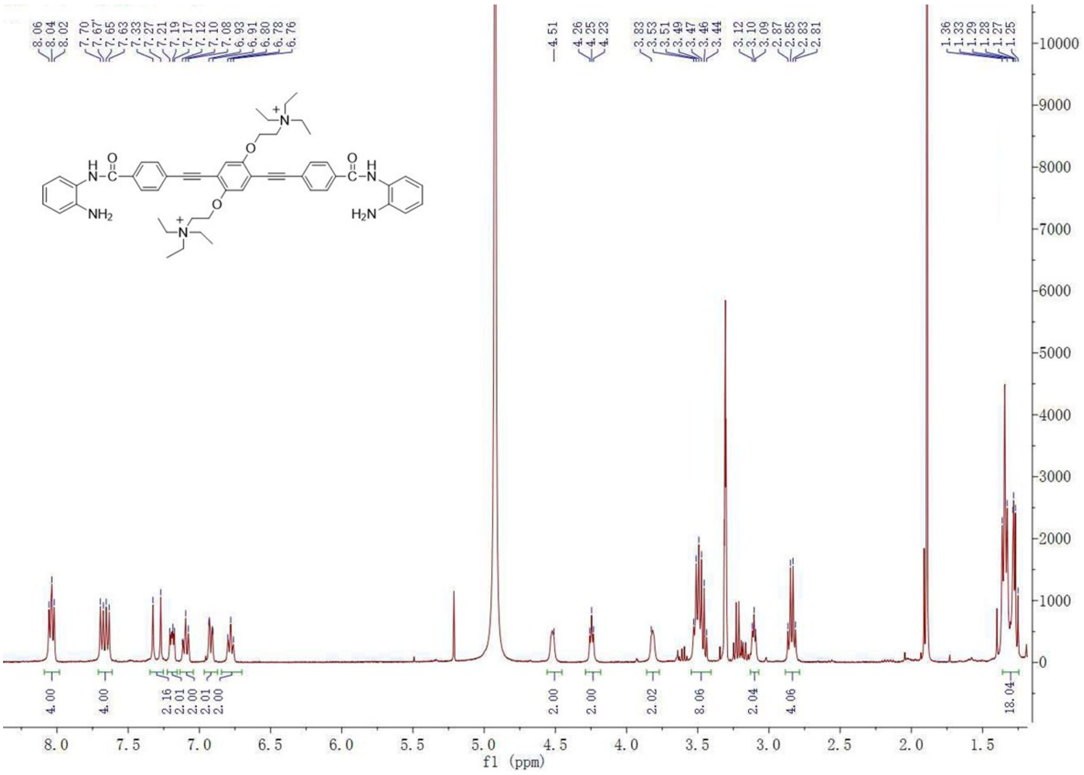

Fig. S7. ^{1}H NMR spectrum (400 MHz, 298 K) of Compound **DBB-NO** in Methanol-d_4 with concentration of 2 mM.

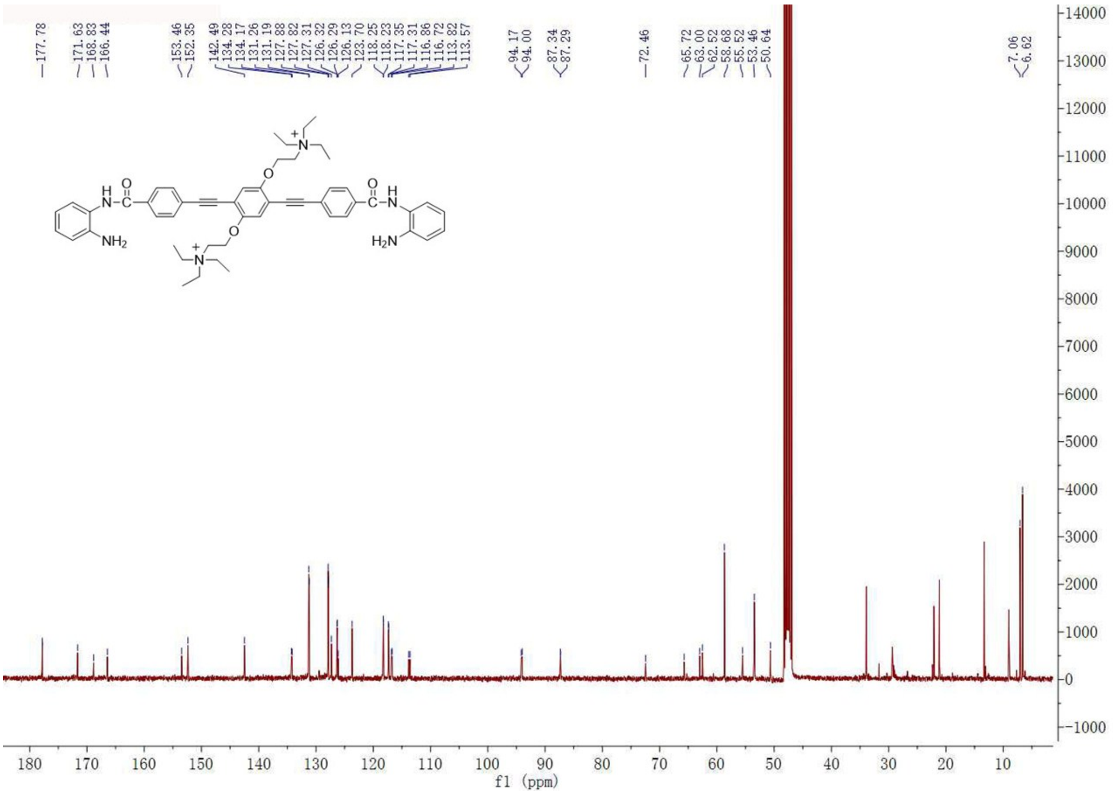

Fig. S8. ^{13}C NMR spectrum (100 MHz, 298 K) of Compound **DBB-NO** in Methanol-d_4 with concentration of 2 mM.

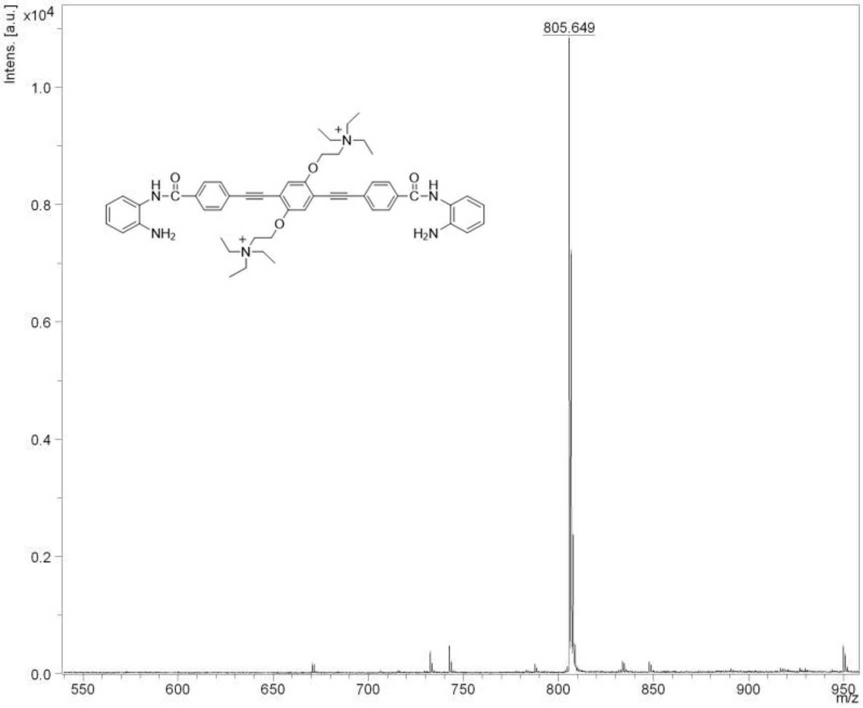

Fig. S9. MALDI-TOF MS chart of Compound **DBB-NO**.

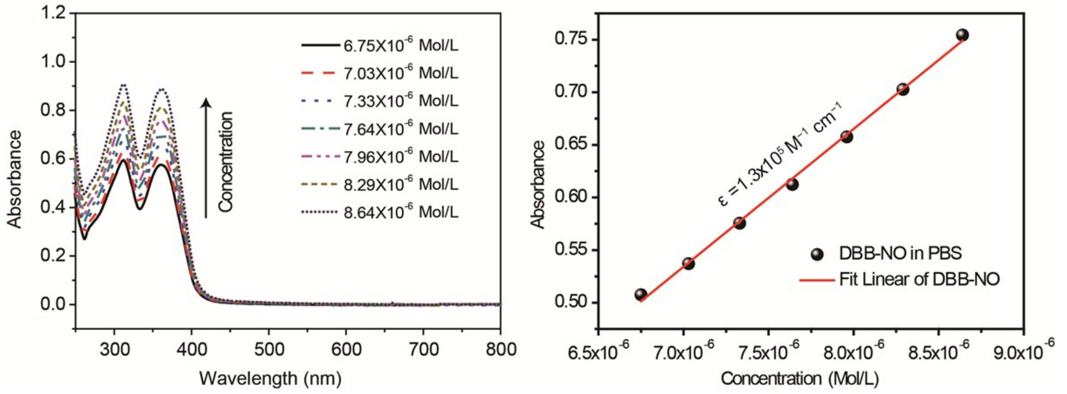

Fig. S10. a) Concentration dependence of the absorption of **DBB-NO** in PBS buffer. b) The plot of optical density at 361 nm versus concentration. The straight line is a linear least-squares fit to the data, indicating an effective molar extinction coefficient (*ε*) of **DBB-NO** at 361 nm.

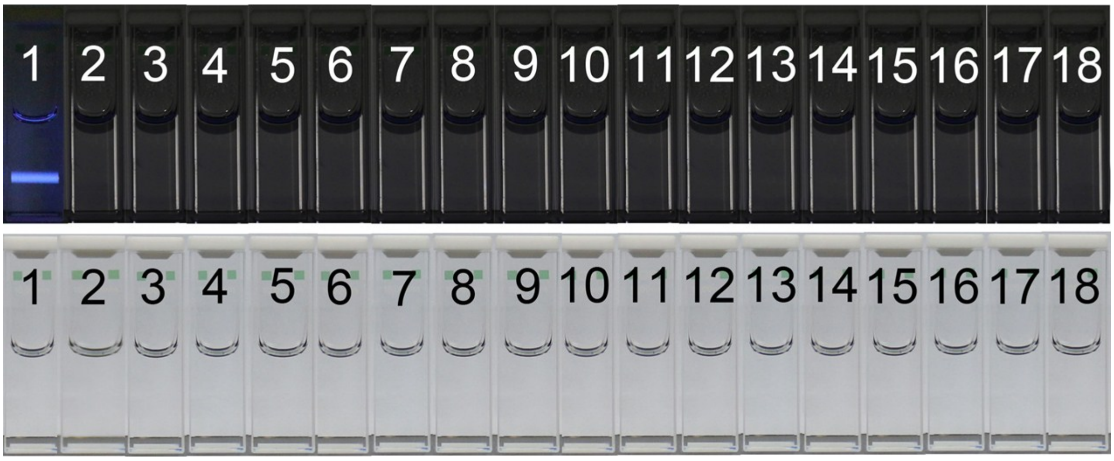

Fig. S11. Digital images of **DBB-NO** (5 μM) upon addition of various competitive species after 15 min under 750 nm femtosecond laser irradiation. (1) DEA•NONOate, (2) **DBB-NO** only, (3) AA, (4) MGO, (5) DHA, (6) NO_2^-, (7) $ONOO^-$, (8) NO_3^-, (9) H_2O_2, (10) HClO, (11) ·OH, (12) $^1O_2$, (13) cysteine (Cys), (14) glutathione (GSH), (15) Fe^{3+}, (16) Ca^{2+}, (17) K^+, (18) Na^+.

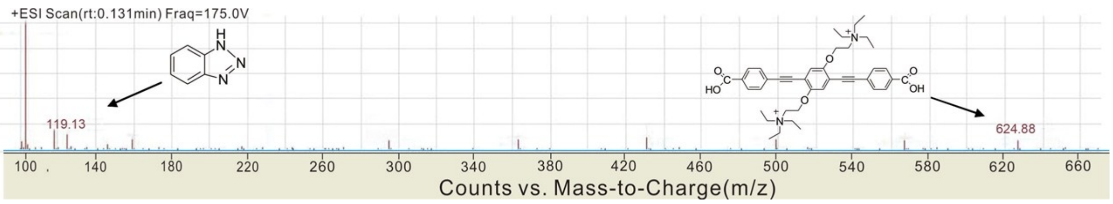

Fig. S12. HRMS of the reaction between **DBB-NO** (5 μM) and 200 equiv DEA·NONOate in PBS buffer for 15 min. The peak at m/z =119.13 is assigned to the product of benzotriazole ($[M+H]^+$= 119). The peaks at m/z 624.88 is assigned to the released **DBB** ($[M-2CH_3]^+$ calcd m/z 624) from **DBB-NO**.

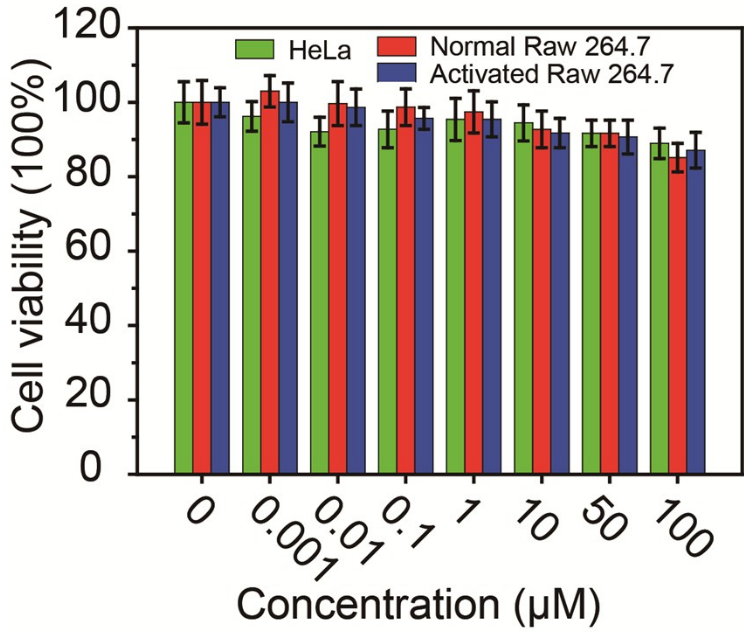

Fig. S13. Dark cytotoxicity of **DBB-NO** in different concentrations without TP-irradiation. No obvious cytotoxicity was observed even the concentrations reach up to 100 μM, suggesting an extremely low dark cytotoxicity of **DBB-NO**.

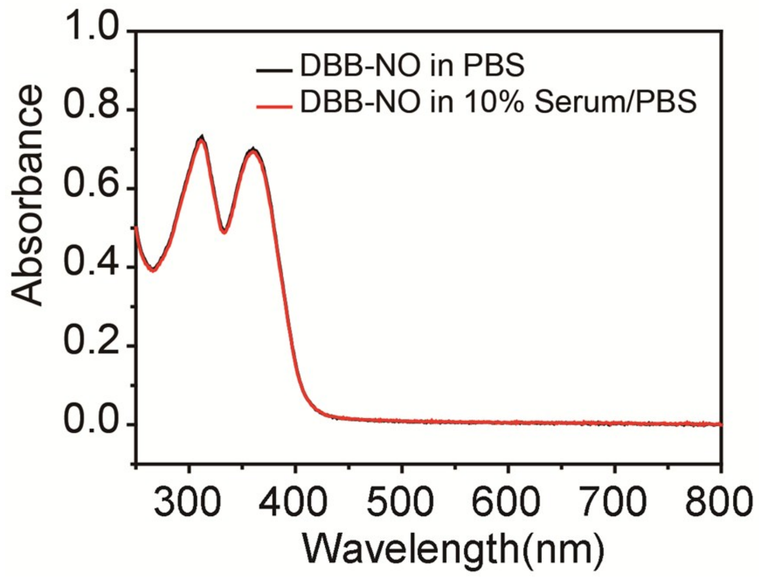

Fig. S14. Absorption spectra of 7.6 μM **DBB-NO** in PBS buffer (pH 7.5) with/without 10% serum, respectively. No obvious spectral changes indicate the excellent chemical stability in physiological environment.

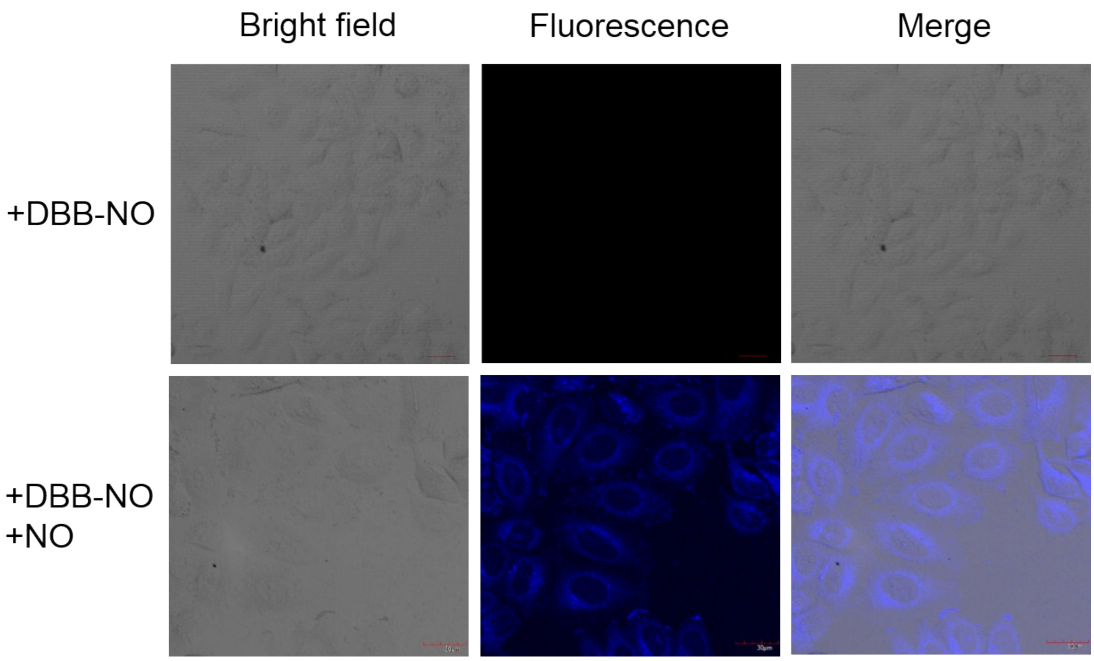

Fig. S15. TP-images of HeLa cells stained with **DBB-NO** in the absence and presence of DEA•NONOate (100 μM, 30 min). Fluorescence was collected at 410-450 nm upon TPE at 750 nm. Scale bar: 30 μm.

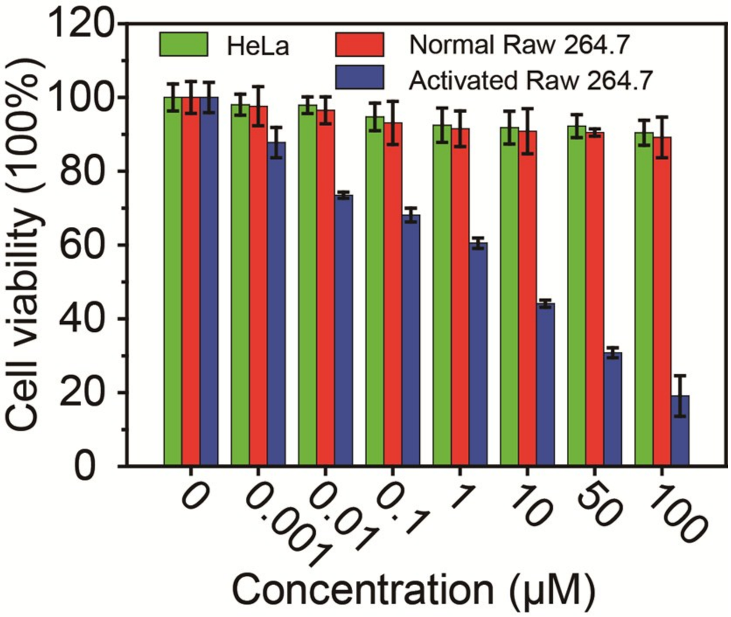

Fig. S16. MTT assay about TP-PDT effect of **DBB-NO**. The viability of HeLa cell and normal macrophage incubated with **DBB-NO** remained nearly 100%, while the viability of activated macrophages incubated with **DBB-NO** was increasingly reduced to 19.1%. This result revealed a NO-activatable TP-PDT of **DBB-NO**.

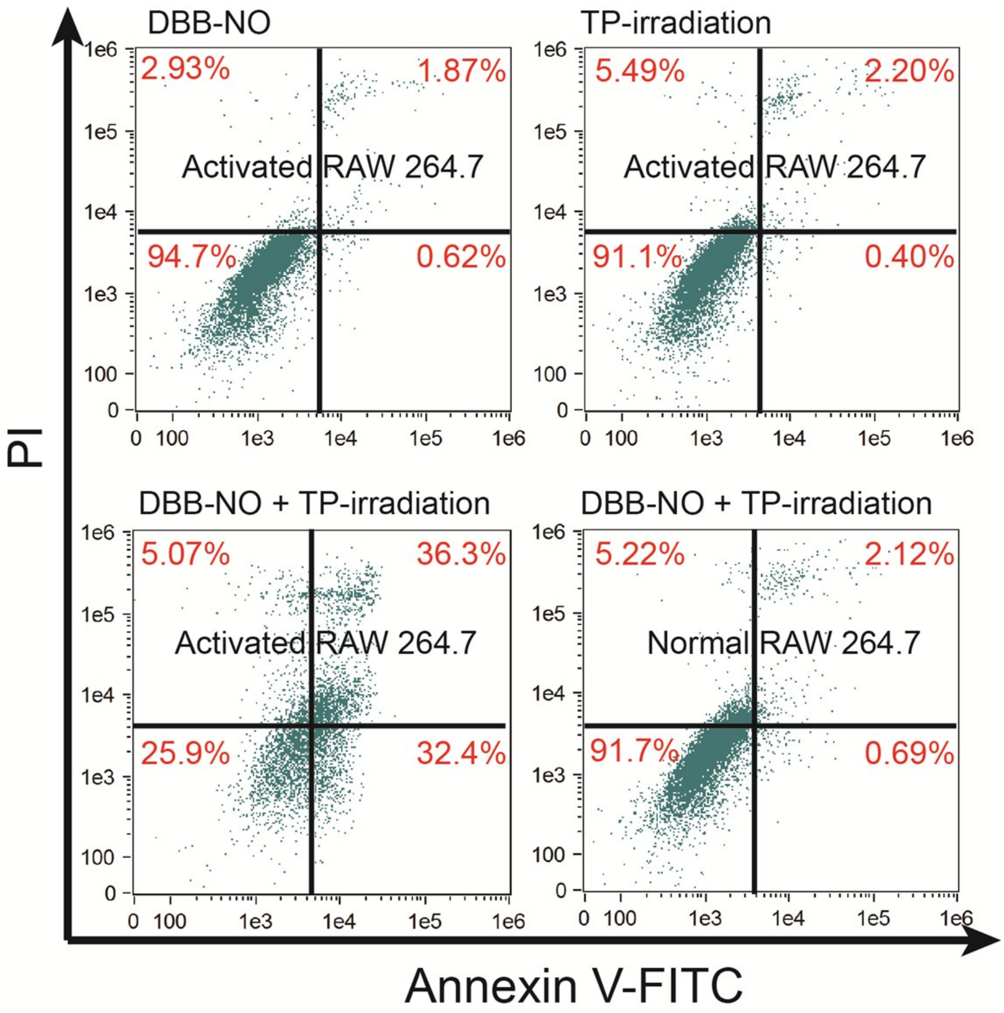

Fig. S17. Flow cytometric assay about TP-PDT effect of **DBB-NO** in which using Annexin FITC/propidium iodide (PI) cell apoptosis to distinguish viable cells from dead cells. The activated macrophages treated with either **DBB-NO** or TP-irradiation alone exhibited a high cell viability (Annexin V-FITC^{-}/PI^{-}) with value exceeding 91.1%. After treated with **DBB-NO** under TP-irradiation, the viable macrophages (Annexin V-FITC^{-}/PI^{-}) decreased to 25.9% and the sum population of early apoptotic (Annexin V-FITC^{+}/PI^{-}) and latest-age apoptotic (Annexin V-FITC^{+}/PI^{+}) macrophages significantly increased (88.7%), indicating a highly efficient TP-PDT. For the control where normal macrophages treated with **DBB-NO** under TP-irradiation, no obvious cell apoptosis was observed. From these results, we concluded that **DBB-NO** is endogenous NO-activatable and can serve as a smart photosensitizer to selectively destroy activated macrophage from normal ones for a precision therapy.

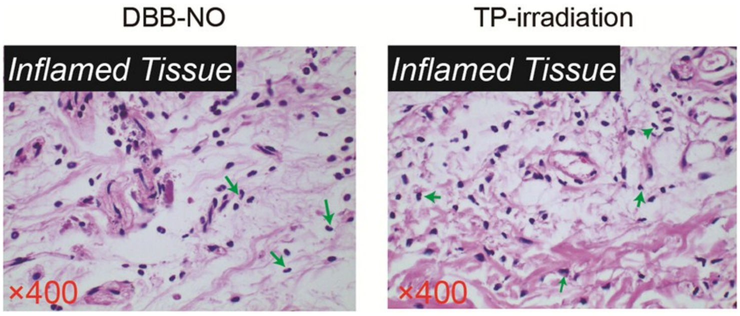

Fig. S18. H&E assay for inflamed mouse to exclude the injury from **DBB-NO** or TP-irradiation alone.

9. References

1. (a) W. B. Hu, T. C. He, R. C. Jiang, J. Yin, L. Li, X. M. Lu, H. Zhao, L. Zhang, L. Huang, H. D. Sun, W. Huang and Q. L. Fan, *Chem. Commun.*, 2017, **53**, 1680; (b) Q. L. Fan, Y. Zhou, X. M. Lu, X. Y. Hou and W. Huang, *Macromolecules*, 2005, **38**, 2927.
2. (a) T. He, Z. B. Lim, L. Ma, H. Li, D. Rajwar, Y. Ying, Z. Di, A. C. Grimsdale and H. Sun, *Chem.--Asian J.*, 2013, **8**, 564; (b) T. He, P. C. Too, R. Chen, S. Chiba and H. Sun, *Chem.--Asian J.*, 2012, **7**, 2090.
3. Y. Q. Sun, J. Liu, H. Zhang, Y. Huo, X. Lv, Y. Shi and W. Guo, *J. Am. Chem. Soc.*, 2014, **136**, 12520.
4. X. Q. Shen, L. Li, A. C. M. Chan, N. Y. Gao, S. Q. Yao and Q. H. Xu, *Adv. Opt. Mater.*, 2013, **1**, 92.
